# SSRP1/SLC3A2 Axis in Arginine Transport: A New Target for Overcoming Immune Evasion and Tumor Progression in Peripheral T‐Cell Lymphoma

**DOI:** 10.1002/advs.202415698

**Published:** 2025-05-08

**Authors:** Yimin Ren, Lei Fan, Ling Wang, Yanping Liu, Jie Zhang, Boya Wang, Ruize Chen, Xiao Chen, Lingyu Zhuang, Yaping Zhang, Handong Sun, Jianyong Li, Wenyu Shi, Hui Jin

**Affiliations:** ^1^ Lymphoma Center, Department of Hematology, The First Affiliated Hospital with Nanjing Medical University Jiangsu Province Hospital Nanjing 210029 China; ^2^ Key Laboratory of Hematology of Nanjing Medical University Nanjing 210029 China; ^3^ Jiangsu Key Lab of Cancer Biomarkers, Prevention and Treatment, Collaborative Innovation Center for Personalized Cancer Medicine Nanjing Medical University Nanjing 210029 China; ^4^ Department of Hematology Affiliated Hospital 2 of Nantong University Nantong 226001 China; ^5^ Wuxi School of Medicine Jiangnan University Wuxi 214122 China; ^6^ Department of Hematology Affiliated Hospital of Nantong University Nantong 226001 China; ^7^ Department of Breast, Women's Hospital of Nanjing Medical University Nanjing Women and Children's Healthcare Hospital Nanjing 210004 China; ^8^ Department of Oncology Affiliated Hospital of Nantong University Nantong 226001 China

**Keywords:** arginine, drug repurposing, immune escape, peripheral T‐cell lymphoma, solute carrier, transcription regulation, tumor progression

## Abstract

Peripheral T‐cell lymphoma (PTCL) is a heterogeneous group of mature T‐cell malignancies with poor prognosis. Therefore, improved therapies are urgently required to improve patient outcomes. In this study, metabolic inhibitor drug screening reveals that quinacrine elicits excellent antitumor activity both in vitro and in vivo by downregulating intracellular arginine levels in PTCL. Single‐cell transcriptomic analyses reveal aberrant arginine metabolism in patients with PTCL, characterized by excessive solute carrier family 3 member 2 (SLC3A2) mediated arginine uptake preferentially in tumor cells. High SLC3A2 expression predicts poor outcomes in PTCL, as SLC3A2‐mediated arginine uptake promotes the malignant behaviors of tumor cells and induces tumor immune escape, thereby fueling tumor progression. Mechanistically, high arginine levels induce global metabolic changes, including enhanced oxidative phosphorylation by promoting nascent RNA synthesis. This work identifies structure‐specific recognition protein 1 (SSRP1), which upregulates SLC3A2, as a co‐transcription factor with JUNB. Quinacrine disrupts SLC3A2‐mediated arginine transport by targeting SSRP1. Combining quinacrine with histone deacetylase inhibitors is a promising therapeutic strategy for PTCL.

## Graphical abstract

Enhanced arginine uptake is a critical feature of metabolic reprogramming in PTCL, characterized by elevated expression of the arginine transporter SLC3A2. SLC3A2‐mediated arginine uptake facilitates PTCL proliferation and survival by enhancing OXPHOS metabolism and inducing immune evasion. Quinacrine downregulates SLC3A2 transcripts by disrupting the SSRP1‐JUNB interaction, thereby offering a promising therapeutic approach for PTCL.

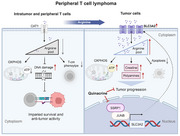

## 1 Introduction

Peripheral T‐cell lymphoma (PTCL), which accounts for approximately 15%–20% of all aggressive non‐Hodgkin's lymphomas, is characterized by the malignant growth of post‐thymic lymphocytes and diverse clinicopathological features.^[^ ^1^ ^]^ PTCL not otherwise specified (PTCL‐NOS), angioimmunoblastic T‐cell lymphoma (AITL), and anaplastic large cell lymphoma (ALCL) are the most common subtypes.^[^ ^2^ ^]^ With the exception of anaplastic lymphoma kinase (ALK) positive ALCL, PTCL generally responds poorly to the standard chemotherapy regimen of cyclophosphamide, doxorubicin, vincristine, and prednisolone (CHOP), leading to a 5‐year survival rate below 30% for most patients.^[^ ^3^, ^4^ ^]^ Therefore, novel therapeutic strategies for this lethal disease are urgently need.

Metabolic reprogramming is a hallmark of cancer development. To sustain their growth and survival in a nutrient‐deficient microenvironment, tumor cells alter their glucose, lipid, and amino acid metabolisms.^[^ ^5^, ^6^ ^]^ Notably, dysregulated amino acid metabolism plays a key role in tumorigenesis and tumor progression,^[^ ^7^, ^8^, ^9^ ^]^ contributing to immune evasion and chemotherapy resistance in tumor cells.^[^ ^10^, ^11^ ^]^ Recent studies have highlighted amino acid metabolic reprogramming as a key driver of lymphoma progression and malignant transformation.^[^ ^12^, ^13^, ^14^, ^15^ ^]^ However, the metabolic landscape of PTCL, particularly regarding amino acid metabolism, remains largely unexplored.

Arginine is a crucial and versatile amino acid involved in various physiological processes, serving as a building block for protein synthesis and a precursor for multiple metabolites, including polyamines, creatine, and nitric oxide.^[^ ^16^, ^17^ ^]^ It is well established that intracellular arginine levels play a critical role in shaping metabolic fitness and supporting the survival of tumor cells.^[^ ^18^, ^19^, ^20^, ^21^ ^]^ Notably, the expression of argininosuccinate synthetase 1 (ASS1), the rate‐limiting enzyme in arginine biosynthesis (the urea cycle), is epigenetically silenced in various malignancies, rendering tumor cells dependent on extracellular arginine.^[^ ^19^, ^20^, ^22^, ^23^ ^]^

Membrane transporters are essential for amino acid trafficking, with solute carriers (SLCs) being considerably upregulated in tumor cells to fulfill metabolic demands.^[^ ^24^, ^25^, ^26^ ^]^ Arginine uptake occurs primarily through three SLC families: SLC7, SLC3, and SLC6.^[^ ^27^ ^]^ Among the proteins in these families, solute carrier family 7 member 1 (SLC7A1) plays a key role in arginine uptake by normal T‐cells, which is crucial for their proliferation, activation, and antitumor functions.^[^ ^28^ ^]^ However, the role of arginine metabolism and its potential transporters in malignant T cells remains unclear, particularly in the context of PTCL, a heterogeneous group of mature, terminally differentiated T‐cell neoplasms.^[^ ^29^ ^]^

Metabolic alterations create collateral vulnerabilities that can be used to identify novel therapeutic targets.^[^ ^30^ ^]^ High‐throughput drug screening (HTS) has been used for drug repurposing and the fast‐tracking of new applications for approved or investigational drugs for multiple cancer types.^[^ ^31^, ^32^, ^33^ ^]^ In this study, we present an HTS strategy based on a metabolic inhibitor drug library aimed to elucidate metabolic vulnerabilities in PTCL. Our results show that quinacrine exhibits potent anti‐PTCL activity both in vitro and in vitro by downregulating intracellular arginine levels. Metabolomic profiling and single‐cell transcriptomic analyses confirm that PTCL exhibits arginine auxotrophy and relies on the arginine transporter solute carrier family 3 member 2 (SLC3A2) for arginine uptake from the environment to sustain proliferation and escape immune surveillance. Furthermore, arginine regulates oncogenic metabolic reprogramming, particularly oxidative phosphorylation (OXPHOS), to promote PTCL progression. Quinacrine, an inhibitor of the facilitation of chromatin transcription (FACT) complex subunit structure‐specific recognition protein 1 (SSRP1), suppresses SLC3A2 transcription, leading to intracellular arginine deficiency and PTCL growth inhibition. Our study highlights the crucial role of arginine transport in PTCL and identifies quinacrine as a promising therapeutic candidate for patients with genetically heterogeneous PTCL.

## 2 Results

### 2.1 In Vitro HTS Identifies Quinacrine as a Potential Drug in Cell Models of PTCL

**Figure 1:**
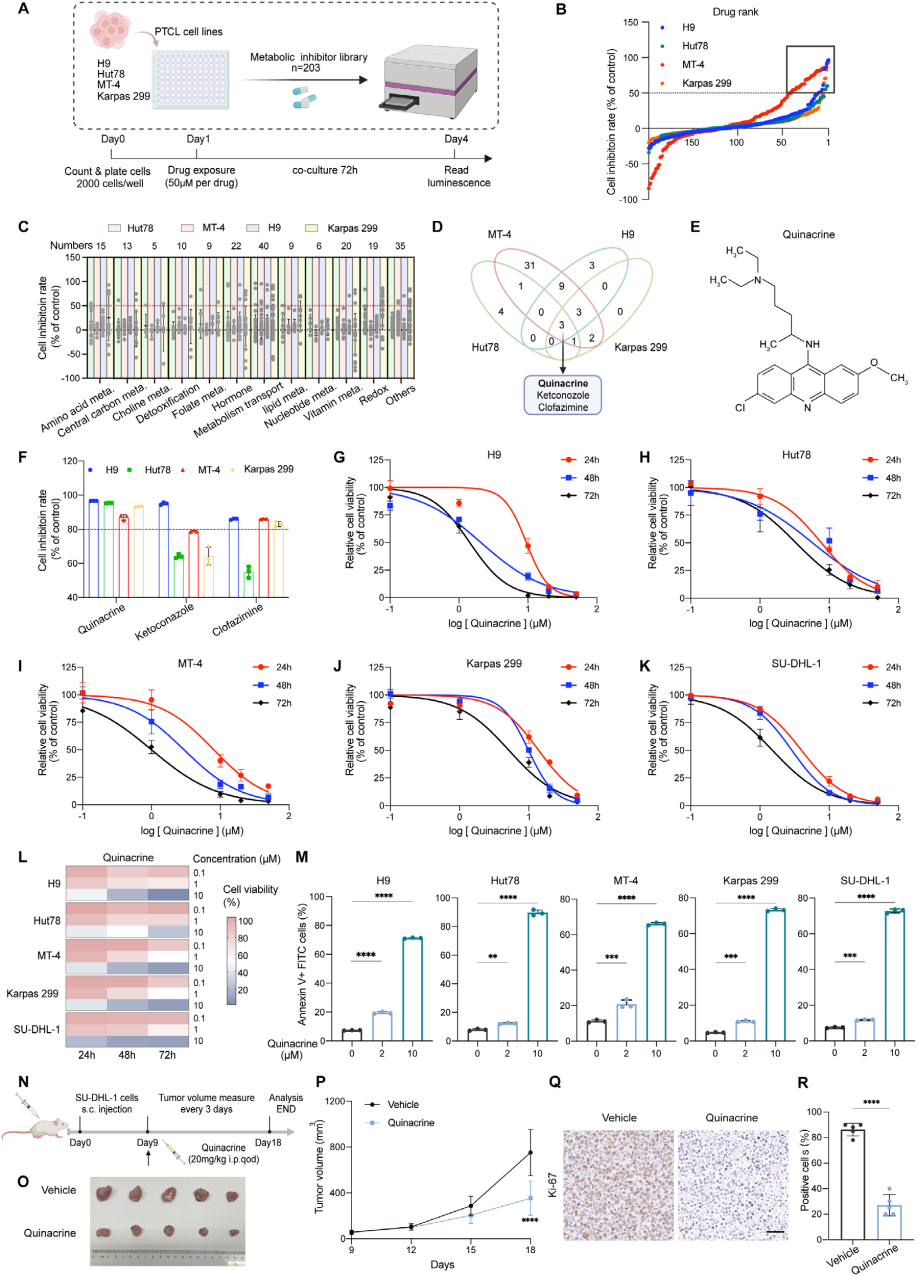
In vitro high‐throughput drug screening (HTS) identifies quinacrine as a potential drug in cell models of peripheral T‐cell lymphoma (PTCL). A) Schematic of HTS in PTCL. B) Waterfall plot of all drugs ranked by cell inhibition rate. Drugs with a >50% cell inhibition rate (dotted line) were categorized as hits (black outline). Four cell models (H9, Hut78, MT‐4, and Karpas 299) are indicated by different colors. C) Scatter plot depicting the pathways targeted by drugs in the metabolic library. The number of drugs targeting different pathways is shown at the top. D) Venn diagram for identifying hits with effectiveness across the four PTCL cell lines. The top hit is bolded. E) Chemical structural formula of quinacrine. F) Drug screening results for the four PTCL cell lines treated with quinacrine, ketoconazole, or clofazimine (*n* = 3). The dotted line represents 0.8. G–K) Relative cell viability of H9 (G), Hut78 (H), MT‐4 (I), Karpas 299 (J), and SU‐DHL‐1 (K) cells treated with different concentrations of quinacrine for 24, 48, or 72 h (*n* = 3). L) Heatmap representation of cell viability for the five PTCL cell lines (G–K) treated with quinacrine using a concentration gradient (0.1 × 10^−6^ m, 1 × 10^−6^ m, and 10 × 10^−6^ m). M) Apoptosis assay of the five PTCL cell lines treated with different concentrations of quinacrine for 48 h (*n* = 3). N) Schematic representation of a SU‐DHL‐1 xenograft mouse model treated with vehicle or quinacrine (*n* = 5). O,P) Photographs of tumors (O) and tumor growth curves (P) in the quinacrine‐ and vehicle‐treated groups (*n* = 5). Q,R) Representative immunohistochemistry (IHC) staining (Q) showing Ki‐67 expression in tumors treated with vehicle or quinacrine and the corresponding quantification (R) (*n* = 5). Scale bar, 50 × 10^−6^ m. For all panels, the data are presented as means ± SD. ^*^ *p* < 0.05; ^**^ *p* < 0.01; ^***^ *p* < 0.001; ^****^ *p* < 0.0001; ns, nonsignificant. For M, *p* values were generated using one‐way ANOVA with multiple comparisons. For P and R, *p* values were generated using Student's two‐tailed unpaired *t*‐test.

Drug repurposing based on HTS is an effective strategy for facilitating precision treatment in which a new indication for an existing compound is identified.^[^ ^34^ ^]^ Here, we used four PTCL cell lines (H9, Hut78, MT‐4, and Karpas 299) as preclinical models to determine drug efficacy and potential metabolic vulnerability in PTCL. The four cell lines were screened against a custom‐curated metabolic inhibitor drug library (**Figure** **1**A and Table S1, Supporting Information). We ranked the drugs based on cell inhibition rate and identified 47 compounds that decreased the fitness of cells by ≥50%, hereafter referred to as “hits” (Figure 1B). These hits inhibit several metabolic processes, including amino acid, lipid, and carbohydrate metabolism (Figure 1C), previously described as metabolic vulnerabilities in lymphoma.^[^ ^13^, ^35^, ^36^ ^]^

Three of the hits (quinacrine, ketoconazole, and clofazimine) exhibited effectiveness across all four cell lines in the preliminary screening (Figure 1D), and quinacrine was the top hit, reducing cell viability to below 20% at the concentration of 50 × 10^−6^ m (Figure 1E,F). To validate the activity of the three hits for PTCL, we subsequently measured the cell viability of five PTCL lines (H9, Hut78, MT‐4, Karpas 299, and SU‐DHL‐1) after treatment with quinacrine, ketoconazole, or clofazimine for 24, 48, and 72 h (Figure 1G–K and Figure S1A–J, Supporting Information). Of the three hits, quinacrine displayed the most robust growth inhibitory effect on all five examined cell lines in a concentration‐ and time‐dependent manner with a half‐maximal inhibitory concentration of 2.08–9.90 × 10^−6^ m for 48 h treatment, as compared to the other two drugs (Figure 1L and Figure S1K, Supporting Information). Quinacrine also induced apoptosis in a concentration‐dependent manner (Figure 1M). Quinacrine is an anti‐inflammatory drug approved for the treatment of malaria and cutaneous lupus erythematosus, and it has been identified as a potential anticancer agent.^[^ ^37^, ^38^ ^]^ Moreover, in light of reports that quinacrine can confer a therapeutic benefit in hematological malignancies,^[^ ^39^, ^40^, ^41^ ^]^ we speculated that it might have greater translation potential in PTCL than the other two drugs. Finally, we assessed the efficacy of quinacrine in vivo using a PTCL xenograft mouse model (Figure 1N). The treatment of xenografts with quinacrine inhibited tumor growth and induced tumor apoptosis (Figure 1O–R, Figures S1L,M, Supporting Information) without affecting mouse body weight (Figure S1N, Supporting Information).

### 2.2 Metabolomic and Transcriptomic Analyses Reveal that Quinacrine Downregulates the Arginine Transporter SLC3A2 and Causes Arginine Deficiency

**Figure 2:**
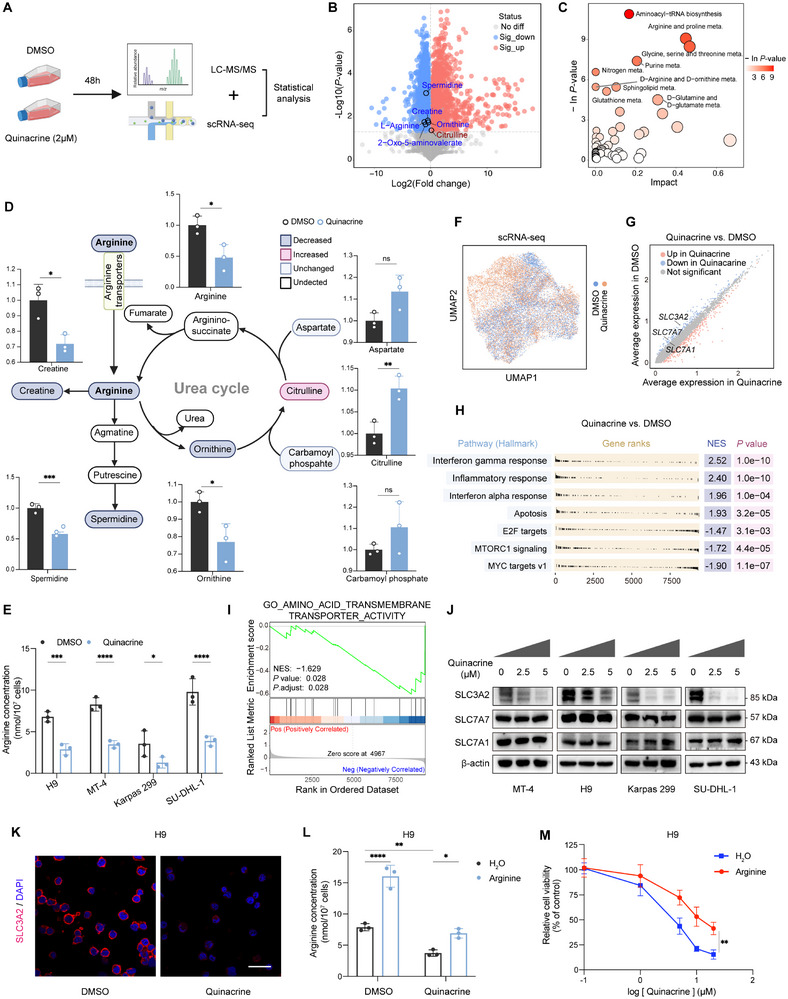
Metabolomic and transcriptomic analyses reveal that quinacrine downregulates the arginine transporter SLC3A2 and causes arginine deficiency. A) Schematic representation of the metabolic and transcript profiling approaches for H9 cells (*n* = 3). DMSO, dimethyl sulfoxide. B) Volcano plot visualization of the log2 fold change (*x*‐axis) and −log10 (*p* value; *y*‐axis) of intracellular metabolites measured by liquid chromatography–mass spectrometry (LC‐MS/MS). Significantly increased (red) or decreased (blue) metabolites related to arginine metabolism are labeled. C) Metabolic pathway analysis plot showing the most strongly impacted metabolic pathways in treated cells, as shown in (A). Pathway impact value computed from MetaboAnalyst topological analysis (*x*‐axis) versus −In *p*‐value obtained from pathway enrichment analysis (*y*‐axis). D) Fold changes in the intracellular metabolite abundance of arginine metabolism detected by LC‐MS/MS in quinacrine‐ and DMSO‐treated cells. The DMSO‐treated levels were set to 1. Significantly increased, decreased, unchanged, and undetected metabolites are color‐labeled as indicated. E) Intracellular arginine concentrations in indicated cell lines treated with quinacrine (2 × 10^−6^ m) for 48 h (*n* = 3). F) Uniform manifold approximation and projection (UMAP) plots for quinacrine‐ (light red) and DMSO‐treated (light blue) H9 cells. G) Volcano plot visualization of differentially expressed genes (DEGs) in H9 cells treated with quinacrine or DMSO for 48 h. Significantly downregulated arginine transporters are labeled. H) Table plot showing hallmark analysis results for significantly enriched pathways in quinacrine‐treated cells vs. control cells. NES, normalized enrichment score. I) Gene Ontology (GO) enrichment analysis plots showing the enriched amino acid transmembrane transporter pathways in quinacrine‐ and DMSO‐treated H9 cells. J) Immunoblots of the indicated proteins in PTCL cell lines treated with different quinacrine concentrations for 48 h (*n* = 3). β‐actin serves as a loading control. K) Representative SLC3A2 immunofluorescence staining of H9 cells treated with quinacrine or DMSO (2 × 10^−6^ m) for 48 h (*n* = 3). Scale bar, 25 × 10^−6^ m. L) Arginine levels in H9 cells treated with quinacrine (2 × 10^−6^ m) or DMSO and cultured in H_2_O or arginine (4 × 10^−3^ m) for 48 h (*n* = 3). M) Relative cell viability of H9 cells treated with different concentrations of quinacrine and cultured in H_2_O or arginine (4 × 10^−3^ m) for 48 h (*n* = 3). For all panels, the data are presented as means ± SD. ^*^ *p* < 0.05; ^**^ *p* < 0.01; ^***^ *p* < 0.001; ^****^ *p* < 0.0001; ns, nonsignificant. For B, D, E, and M, *p* values were generated using Student's two‐tailed unpaired *t*‐test. For L, *p* values were generated using a two‐way ANOVA with multiple comparisons. For I, *p* values were generated using a permutation test.

Quinacrine was previously identified as a nonspecific inhibitor of phospholipase A2, which converts membrane phospholipids into arachidonic acid.^[^ ^42^ ^]^ However, the addition of arachidonic acid to the culture medium was unable to rescue the proliferation of PTCL cells treated with quinacrine (Figure S2A–E, Supporting Information), indicating that phospholipase A2 inhibition may not be the underlying mechanism of action of quinacrine in PTCL. To gain insights into the crucial metabolic pathways remodeled by quinacrine, we performed liquid chromatography–mass spectrometry (LC‐MS/MS) analyses of H9 cells (**Figure** **2**A). Pronounced differences in intracellular metabolites were observed, as revealed by principal component analysis (PCA) (Figure S2F, Supporting Information). A total of 84 upregulated and 167 downregulated metabolites were identified (Figure 2B). The most striking changes were the depletion of arginine and arginine metabolism intermediates, including creatine, spermidine, and ornithine, in quinacrine‐treated cells (Figure 2B,D). Metabolite set enrichment analysis also showed a downregulated abundance of metabolites belonging to arginine and proline, arginine and ornithine, nitrogen, and purine metabolism (Figure 2C), indicating the substantial role of altered arginine metabolism in cells treated with quinacrine. To confirm the effects of quinacrine on arginine, we measured the arginine levels in PTCL cells. In agreement with the aforementioned findings, arginine levels decreased in all PTCL cell lines upon quinacrine treatment (Figure 2E).

To cross‐compare quinacrine‐mediated transcriptional and metabolic changes within cells, single‐cell RNA sequencing (scRNA‐seq) was performed in parallel with LC‐MS/MS (Figure 2F), followed by the mapping of well‐characterized metabolic genes in the arginine metabolic network (Figure S2G, Supporting Information). Differentially expressed gene (DEG) analysis based on scRNA‐seq revealed that solute carrier family transporters (SLC3A2, SLC7A1, and SLC7A7), which mediate arginine uptake, were transcriptionally downregulated in the quinacrine‐treated group (Figure 2G). Few changes were observed in the abundance of mRNAs encoding arginine biosynthesis‐ and catabolism‐related genes (Figure S2I, Supporting Information). Immunoblot analysis confirmed that quinacrine did not affect the expression of key enzymes involved in the arginine catabolic pathway (Figure S2J, Supporting Information). However, quinacrine treatment led to the induction of the apoptotic pathway and the inhibition of pathways associated with cell proliferation, such as E2F and MYC targets, consistent with the preceding results (Figure 2H). Notably, mTORC1 inhibition may result from arginine deficiency, which is a part of the cellular integrated stress response.^[^ ^43^, ^44^ ^]^ Gene Ontology (GO) enrichment analyses of biological processes revealed that amino acid transmembrane transporter activity was significantly downregulated with quinacrine treatment (Figure 2I), suggesting that arginine transport was a primarily altered metabolic pathway remodeled by quinacrine. Although significant downregulation was observed in the mRNA levels of SLC3A2, SLC7A1, and SLC7A7, only the protein expression of SLC3A2 was consistently diminished in a dose‐dependent manner across the PTCL cell lines upon quinacrine treatment (Figure 2J,K, and Figure S2K, Supporting Information). Moreover, quinacrine treatment did not induce the compensatory expression of SLC7A1 or SLC7A7, suggesting that SLC3A2 may be responsible for quinacrine‐induced arginine deficiency as a crucial arginine transporter.

To further test the role of quinacrine in PTCL arginine transport, we added arginine to H9 and Karpas 299 cell supernatants, in which the concentration of arginine increased 4‐fold compared with that in the standard medium (containing 1 × 10^−3^ m arginine). Supplementation with a supraphysiological concentration of arginine appreciably increased intracellular arginine levels and partially weakened the effect of quinacrine on tumor cell viability (Figure 2L,M and Figure S2L,M, Supporting Information). This indicated that quinacrine plays an obligatory role in regulating the intracellular arginine pool, leading to the inhibition of PTCL cell growth.

### 2.3 scRNA‐seq Analysis Reveals Aberrant Arginine Uptake in Patients with PTCL

To characterize arginine metabolism in patients with PTCL, we analyzed a scRNA‐seq dataset produced at our center (Group1) based on lymph node biopsies from eight patients with AITL, a prominent PTCL subtype. Distinct cell types in the tumor microenvironment (TME) and T follicular helper (Tfh) tumor cells, the origin of AITL, were identified according to our previous study (Figure S3A, Supporting Information).^[^ ^45^ ^]^ We first analyzed the single‐cell expression of ASS1, ornithine transcarbamoylase (OTC), arginase2 (ARG2), and argininosuccinate lyase (ASL), four key enzymes in the urea cycle that produce arginine during the detoxification of excess ammonium,^[^ ^46^ ^]^ as shown in Figure S3B (Supporting Information). Consistent with normal T cells,^[^ ^47^ ^]^ the arginine biosynthesis rate‐limiting enzyme *ASS1* was transcriptionally silenced in T‐cell subsets of AITL. The arginine biosynthesis pathway scores of T cells, based on the relevant Kyoto Encyclopedia of Genes and Genomes (KEGG) pathway (has00220), further confirmed that the urea cycle was suppressed in AITL cells (Figure S3C, Supporting Information). The suppression of the urea cycle makes arginine auxotrophic tumor cells dependent on extracellular arginine.

**Figure 3:**
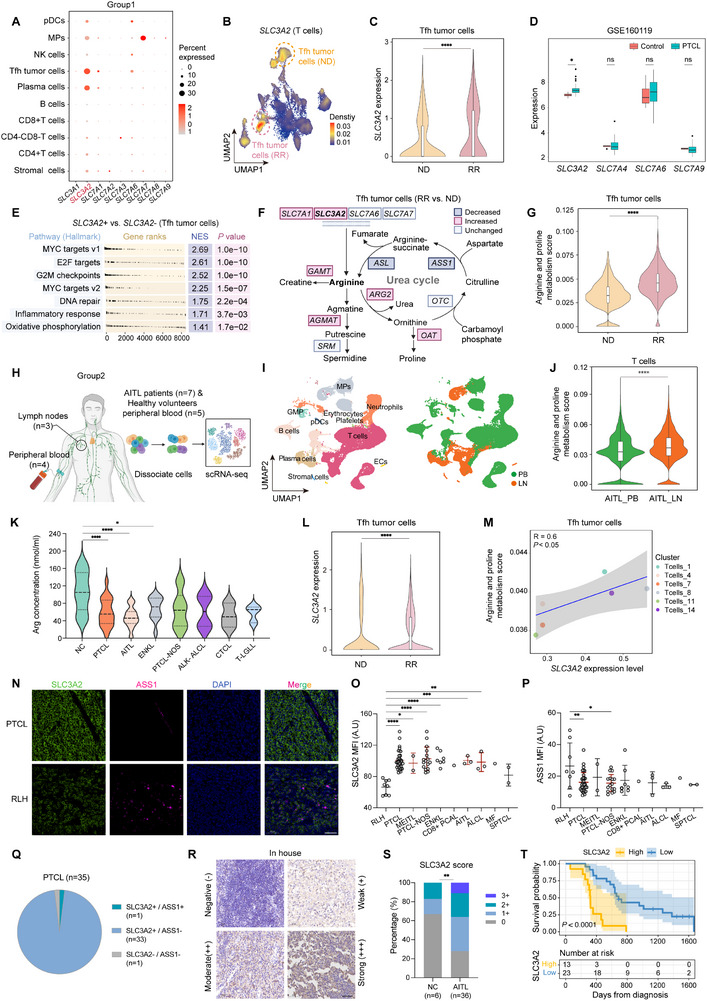
Characterization of arginine metabolism in patients with peripheral T‐cell lymphoma (PTCL). A) Dot plot of arginine transporter expression in the indicated cell types from Group1. pDCs, plasmacytoid dendritic cells; MPs, mononuclear phagocytes; NK cells, natural killer cells; Tfh, follicular helper T cells. B) Density plot of *SLC3A2* expression shown in the uniform manifold approximation and projection (UMAP) visualization of T cells. Tfh tumor cells are indicated by dashed circles. C) Violin plot showing *SLC3A2* expression in Tfh tumor cells from the newly diagnosed (ND) and relapsed/refractory (RR) groups. D) Box plot of the expression of arginine transporters *SLC3A2*, *SLC7A4*, *SLC7A6*, and *SLC7A9* in control and PTCL patients from GSE160119. E) Table plot showing hallmark analysis results of significantly enriched pathways in *SLC3A2*+ Tfh tumor cells vs. *SLC3A2*− Tfh tumor cells. NES, normalized enrichment score. F) Schematic representation of arginine metabolism. Changes in the mRNA levels of enzymes and transporters in Tfh tumor cells from the RR group compared to those in the ND group are shown. Color‐coding represents the level of log2‐fold change as indicated. G) Violin plot showing the arginine and proline metabolism scores based on KEGG pathway enrichment (has00330) in Tfh tumor cells from the ND and RR groups. H) Schematic of the scRNA‐seq design for Group2. I) UMAP of 11 cell subtypes (left) and two groups (PBMC and LN) (right) identified in 12 specimens. J) Violin plot showing arginine and proline metabolism scores based on KEGG pathway enrichment (has00330) in T cells from the lymph nodes and peripheral blood of patients with angioimmunoblastic T‐cell lymphoma (AITL). K) Arginine levels in the plasma of 15 normal controls (NCs) and 44 PTCL patients. The 44 patients included those with AITL (*n* = 12), extranodal NK/T‐cell lymphoma (ENKL) (*n* = 12), PTCL‐NOS (*n* = 10), ALK‐ ALCL (*n* = 4), cutaneous T‐cell lymphoma (CTCL) (*n* = 3), and T‐cell large granular lymphocyte leukemia (T‐LGLL) (*n* = 3). L) Violin plot showing *SLC3A2* expression in Tfh tumor cells from the ND and RR groups. M) Scatter plot showing the correlation between *SLC3A2* expression and arginine and proline metabolism scores in Tfh tumor cell clusters. N) Representative SLC3A2 (green) and ASS1 (red) immunofluorescence staining in a PTCL tissue microarray containing 35 PTCL and eight reactive lymphoid hyperplasia (RLH) samples. Scale bar, 50 × 10^−6^ m. O,P) Mean fluorescence intensity (MFI) of SLC3A2 (O) and ASS1 (P) in the PTCL tissue microarray. Q) Pie chart showing the percentage of three phenotypes (SLC3A2+/ASS1+, SLC3A2+/ASS1−, and SLC3A2−/ASS1−) in the PTCL samples. R) Patterns of different levels of SLC3A2 expression in normal lymph nodes and AITL tissues, detected using immunohistochemistry (IHC) analysis. Scale bar, 50 × 10^−6^ m. S) Distribution of SLC3A2 expression in tissues from 36 patients with AITL and 6 NCs. T) Overall survival (OS) of patients with AITL based on SLC3A2 expression. For all panels, the data are presented as means ± SD. ^*^ *p* < 0.05; ^**^ *p* < 0.01; ^***^ *p* < 0.001; ^****^ *p* < 0.0001; ns, nonsignificant. For C, D, G, J, and L, *p* values were generated using the Wilcoxon rank‐sum test with default parameters. For E, *p* values were generated using a permutation test. For K, O, and P, *p* values were generated using one‐way ANOVA with multiple comparisons. For M, *p* value was generated using Pearson's test. For S, *p* value was generated using Fisher's exact test. For T, *p* value was generated using the log‐rank test.

Next, we analyzed the expression of the SLC family members implicated in arginine transport in distinct cell types (**Figure** **3**A). *SLC3A2* transcripts were highly and preferentially expressed in Tfh tumor cells (Figure 3A,B, and Figure S3D, Supporting Information). Moreover, *SLC3A2* expression increased during progression, as Tfh tumor cells in the relapsed/refractory (RR) group expressed higher levels of *SLC3A2* than those in the newly diagnosed (ND) group (Figure 3B,C). Similarly, among SLC family members, only *SLC3A2* was considerably upregulated in PTCL samples relative to normal lymph node samples in the GSE160119 dataset (Figure 3D). In addition, hallmark analysis of DEGs showed that *SLC3A2*+ Tfh tumor cells were particularly enriched in pathways closely associated with cell proliferation, inflammatory responses, and oxidative phosphorylation (Figure 3E and Figure S3E, Supporting Information), indicating that *SLC3A2* expression is related to AITL progression. *SLC3A2* expression was correlated with cell survival and proliferation in the GSE160119 dataset (Figure S3F, Supporting Information). These results suggest a putative role for SLC3A2 in selectively directing arginine uptake and regulating malignant biological behaviors in tumor cells, but not in normal or TME T cells.

We analyzed the expression of key enzymes involved in the arginine catabolism pathway, and found that guanidinoacetate methyltransferase (GAMT), agmatinase (AGMAT), ARG2, and ornithine aminotransferase (OAT) levels, as well as arginine metabolism pathway scores of Tfh tumor cells, were notably higher in the RR group than in the ND group (Figure 3F,G, and Figure S3G–I, Supporting Information). This further indicates that arginine metabolism is upregulated during tumor progression.

To further delineate the arginine distribution in PTCL, we collected lymph node samples from three patients with AITL and peripheral blood samples from four patients with AITL and five healthy volunteers (Group2) for scRNA‐seq (Figure 3H and Table S2, Supporting Information). Eleven specific cell types were identified using signature genes (Figure 3I, Figure S3J,K, Supporting Information). Lymph node T cells had higher arginine metabolism scores than peripheral blood T cells (Figure 3J). Moreover, the average plasma concentration of arginine was lower in patients with PTCL than in healthy volunteers (Figure 3K), whereas arginine levels were elevated in PTCL cell lines compared to those in normal T cells (Figure S3M, Supporting Information). These findings suggested that tumor cells overconsume arginine from the environment, leading to arginine deficiency in the circulation. In addition, we confirmed that *SLC3A2* levels increased with tumor progression in Group2 (Figure 3L and Figure S3L, Supporting Information). In addition, *SLC3A2* levels were positively associated with arginine metabolism pathway scores in Tfh tumor cells (Figure 3M).

PTCL is characterized by highly heterogeneous clinical manifestations.^[^ ^48^ ^]^ To further determine the clinical and pathological relevance of SLC3A2 and the suppression of ASS1 across different PTCL subtypes, we performed immunofluorescence to detect SLC3A2 and ASS1 expression in a tissue microarray containing 35 PTCL and eight reactive lymphoid hyperplasia (RLH) samples (Figure 3N). The distribution of PTCL subtypes is summarized in Figure S3N (Supporting Information), and the clinicopathological characteristics of the samples in the tissue microarray are listed in Table S3 (Supporting Information). As expected, SLC3A2 was expressed in almost all samples, whereas ASS1 was undetectable in 32 samples and was weakly expressed in only one sample (Figure 3P,Q). SLC3A2 expression was significantly higher in PTCL than in RLH (Figure 3O).

Moreover, we assembled a cohort of 36 patients with AITL and six healthy volunteers (Table S2, Supporting Information). Immunohistochemistry (IHC) analysis of samples from this cohort showed that SLC3A2 expression was significantly higher in AITL than in normal lymph nodes (Figure 3R and Figure 3S, Supporting Information). Kaplan–Meier survival analysis revealed that increased SLC3A2 expression was significantly associated with shorter overall survival (OS) (*p* < 0.0001) in patients with AITL (Figure 3T). Finally, we evaluated the protein levels of several arginine transporters and key enzymes in the arginine metabolism pathway in the five PTCL cell lines (Figure S2H, Supporting Information). Immunoblotting showed that SLC3A2 levels were much higher in all cell lines than in normal T cells isolated from healthy volunteers, and nearly all cell lines exhibited low or undetectable ASS1 levels (Figure S2H, Supporting Information), indicating that arginine uptake by arginine transporters is a major arginine source in PTCL cell lines. Collectively, these results suggest that arginine uptake is overly active in PTCL tumor cells and that SLC3A2, a putative arginine transporter, is highly expressed in patients with PTCL and predicts poor prognosis.

### 2.4 PTCL Relies on SLC3A2‐Mediated Arginine Uptake to Ensure Proliferation and Escape Immune Surveillance

**Figure 4:**
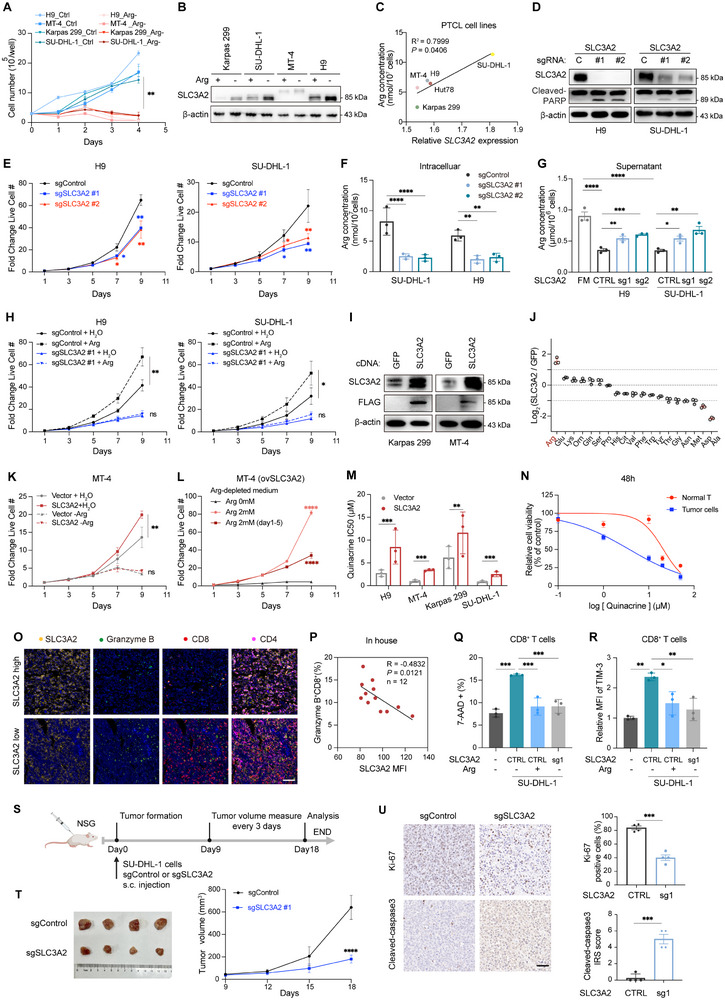
Peripheral T‐cell lymphoma (PTCL) relies on SLC3A2‐mediated arginine uptake to proliferate. A) Growth curves of PTCL cell lines under control and arginine‐depleted (‐Arg) conditions (*n* = 3). B) Immunoblots of SLC3A2 protein in PTCL cell lines under control and arginine‐restricted conditions (*n* = 3). β‐actin serves as a loading control. C) Scatter plot showing the correlation between *SLC3A2* expression and arginine levels in PTCL cell lines (*n* = 3). D) Immunoblots of Cas9+ H9 and SU‐DHL‐1 cells expressing nontargeting control or independent sgRNAs against *SLC3A2*. Whole‐cell lysates were obtained on day 7 after sgRNA expression (*n* = 3). β‐actin serves as a loading control. E) Growth curves of Cas9+ H9 (left) and SU‐DHL‐1 (right) cells expressing nontargeting control or independent sgRNAs targeting *SLC3A2*. Analysis was performed 96 h after sgRNA expression and 48 h after puromycin selection (*n* = 3). F,G) Arginine levels in the cell lysates (F) and supernatants (G) of the indicated cells. FM, fresh medium (*n* = 3). H) Growth curves of H9 (left) and SU‐DHL‐1 (right) cells after the indicated treatments (*n* = 3). I) Immunoblots of whole‐cell lysates from Karpas 299 and MT‐4 cells with control green fluorescent protein (GFP) or SLC3A2‐FLAG cDNA 7 days after cDNA expression (*n* = 3). β‐actin serves as a loading control. J) Fold changes in intracellular amino acid levels detected by liquid chromatography–mass spectrometry (LC‐MS) in Karpas 299 cells expressing control or *SLC3A2* sgRNA (*n* = 3). K,L) Growth curves of MT‐4 cells expressing a vector (control) or SLC3A2 cDNA after the indicated treatments (*n* = 3). M) Half‐maximal inhibitory concentration in PTCL cells expressing a vector (control) or SLC3A2 cDNA treated with different concentrations of quinacrine for 48 h. N) Relative viability of normal T cells isolated from healthy volunteers and tumor cells isolated from patients with angioimmunoblastic T‐cell lymphoma (AITL) treated with different concentrations of quinacrine for 24 h (*n* = 3). O,P) Representative staining for SLC3A2, granzyme B, CD8, and CD4 in AITL tissue (O). Scale bar, 100 × 10^−6^ m. Correlation between SLC3A2 expression and the percentage of granzyme B^+^CD8^+^ cells is shown (P) (*n* = 12). Q,R) Flow cytometric analysis of 7‐AAD‐positive CD8^+^ T cells (Q) and relative mean fluorescence intensity (MFI) of TIM‐3 on CD8^+^ T cells (R) cocultured with SLC3A2‐WT or SLC3A2‐ knockout SU‐DHL‐1 cells and/or arginine addition (*n* = 3). S) Schematic representation of a xenograft mouse model constructed using Cas9+ SU‐DHL‐1 cells expressing a nontargeting control or an sgRNA targeting *SLC3A2*. T) Photographs of tumors (left) and tumor growth curves (right) of SLC3A2‐WT and SLC3A2‐knockout groups in the SU‐DHL‐1 xenograft mice (*n* = 4). U) Representative immunohistochemistry (IHC) staining showing Ki‐67 and cleaved‐caspase3 expression in the subcutaneous tumors of the SU‐DHL‐1 xenograft mouse model expressing control or *SLC3A2* sgRNA (left) and the corresponding quantification (right) (*n* = 4). Scale bar, 50 × 10^−6^ m. For all panels, the data are presented as means ± SD. ^*^ *p* < 0.05; ^**^ *p* < 0.01; ^***^ *p* < 0.001; ^****^ *p* < 0.0001; ns, nonsignificant. For E–H, K, L, Q, and R, *p* values were generated using one‐way ANOVA with multiple comparisons. For C and P, *p* values were generated using Pearson's test. For A, J, M, T, and U, *p* values were generated using Student's two‐tailed unpaired t‐test.

Given these results, we investigated whether high arginine levels were critical for PTCL proliferation, survival, or both. Four‐day proliferation assays indicated that PTCL cell growth was severely impaired when the cells were deprived of exogenous arginine (**Figure** **4**A). Arginine deprivation increased Annexin V uptake by PTCL cells (Figure S4A, Supporting Information), indicating the induction of apoptosis. Cell cycle analysis also demonstrated that arginine deprivation inhibited tumor growth, with cell cycle arrest most pronounced at the G1 stage (Figure S4B, Supporting Information). We predicted that SLC3A2 is an essential transporter under nutrient‐limited conditions. As expected, SLC3A2 expression was significantly upregulated during arginine deprivation (Figure 4B). Moreover, *SLC3A2* expression was positively correlated with intracellular arginine levels in PTCL cell lines (Figure 4C). Considering that SU‐DHL‐1 cells exhibited the highest levels of arginine and SLC3A2 expression, we ultimately selected H9, Karpas 299, MT‐4, and SU‐DHL‐1 cell lines for further study.

To determine the functional relevance of SLC3A2 in PTCL and characterize the effects of acute SLC3A2 loss‐of‐function on PTCL proliferation, CRISPR mutagenesis was performed with SLC3A2 targeting sgRNAs (Figure 4D). SLC3A2 depletion significantly impaired the growth of PTCL cells (H9 and SU‐DHL‐1) (Figure 4E). To examine the effects of SLC3A2 depletion on cell survival, apoptosis assays were performed on PTCL cells 7 days after sgRNA expression and puromycin selection, the time point at which control and SLC3A2‐depleted cell growth curves diverged. The loss of SLC3A2 significantly promoted apoptosis, as confirmed by PARP cleavage and flow cytometry (Figure 4D and Figure S4C, Supporting Information). Conversely, lentiviral transduction‐mediated SLC3A2 overexpression in Karpas 299 and MT‐4 cells promoted cell proliferation and inhibited apoptosis (Figure 4I and Figure S4D, Supporting Information). These findings indicate that SLC3A2 facilitates PTCL proliferation.

Subsequently, we investigated whether the tumor‐promoting effects of SLC3A2 were mediated by arginine uptake. SLC3A2 depletion increased arginine in the culture medium and decreased intracellular arginine levels (Figure 4F,G), while its overexpression increased extracellular arginine consumption and intracellular arginine levels (Figure S4E,F, Supporting Information). Cytoplasmic amino acid quantification also confirmed that arginine, but not other SLC3A2 substrates, was significantly upregulated in Karpas 299 cells transfected with the SLC3A2 vector compared to its levels in the control vector (Figure 4J), indicating that SLC3A2 selectively mediates arginine transport in PTCL. Moreover, SLC3A2 depletion abrogated the difference in proliferation between arginine‐treated and control cells (Figure 4H). The pro‐tumoral effect of SLC3A2 overexpression was also abolished in PTCL cells cultured in medium lacking arginine (Figure 4K and Figure S4G, Supporting Information); however, adding arginine to the medium fully rescued the SLC3A2 overexpression‐induced decrease in PTCL cell proliferation. The withdrawal of exogenous arginine on day 5 of the proliferation assay rapidly inhibited the growth of the PTCL cells (Figure 4L and Figure S4H, Supporting Information). These findings indicate that arginine stimulates the growth of PTCL cells in an SLC3A2‐dependent manner. Notably, SLC3A2 overexpression markedly decreased the sensitivity of PTCL cells to quinacrine (Figure 4M). It identified that quinacrine downregulated the intracellular arginine pool by targeting SLC3A2, an essential arginine transporter in PTCL.

SLC7A1 is primarily responsible for arginine uptake by human T cells.^[^ ^28^ ^]^ Accordingly, we investigated the potential role of SLC3A2 in facilitating arginine uptake by normal T cells. Interestingly, SLC3A2 depletion had no significant effect on proliferation or arginine levels in CD8^+^ T cells isolated from healthy donor peripheral blood mononuclear cells (PBMCs), likely due to the compensatory effect of SLC7A1 on cell survival (Figure S4I–M, Supporting Information). Likewise, normal T cells were less sensitive to quinacrine compared to tumor cells (Figure 4N).

Arginine is recognized for its role in enhancing T‐cell survival through interactions with nuclear proteins, as well as its ability to augment antitumor activity by facilitating the generation of central memory‐like T cells.^[^ ^47^ ^]^ Consistent with previous studies,^[^ ^49^, ^50^, ^51^, ^52^ ^]^ arginine deprivation markedly inhibited CD8^+^ T‐cell proliferation and enhanced apoptosis (Figure S4N,O, Supporting Information). Since arginine deficiency occurred in the blood circulation of patients with PTCL (Figure 3J,K), we speculated that PTCL cells overconsume arginine, outcompeting T cells and impairing antitumor immunity by overexpressing SLC3A2. Multicolor immunofluorescence analyses of AITL samples revealed that SLC3A2 expression in tumor cells was inversely correlated with granzyme B^+^CD8^+^ T‐cell infiltration (Figure 4O,P). To mimic in vivo conditions, we cocultured CD8^+^ T cells with SLC3A2 wild‐type SU‐DHL‐1 cells and observed increased expression of 7‐AAD, TIM‐3, and PD‐1 on CD8^+^ T cells, which was reversed by arginine addition. Coculturing with SLC3A2‐knockout SU‐DHL‐1 cells attenuated the increase in 7‐AAD, TIM‐3, and PD‐1 expression (Figure 4Q,R, and Figure S4P, Supporting Information). However, coculturing with SLC3A2‐overexpression Karpas 299 cells induced the increase in 7‐AAD, TIM‐3, and PD‐1 expression on CD8^+^ T cells, which was again mitigated by arginine addition (Figure S4Q–S, Supporting Information). These results suggest that PTCL cells overconsume arginine in the environment by overexpressing SLC3A2, which impairs CD8^+^ T‐cell survival and function. Indeed, the results highlight quinacrine as an attractive therapeutic option to avoid the undesirable side effects of T‐cell inhibition.

Finally, we examined the role of SLC3A2 in vivo (Figure 4S). SLC3A2 loss impaired tumor growth without affecting body weight (Figure 4T,U, and Figure S4V, Supporting Information). *SLC3A2* sgRNAs maintained reduced SLC3A2 levels and lower endpoint intratumoral arginine levels (Figure S4T,U, Supporting Information). Together, these results indicate that PTCL cells rely on SLC3A2‐mediated arginine uptake to ensure proliferation and escape immune surveillance.

### 2.5 Single‐Cell Dynamic Sequencing Reveals that Arginine Deficiency Reduces Oxidative Phosphorylation (OXPHOS) and Induces Nascent RNA Synthesis of SLC3A2 via JUNB

**Figure 5:**
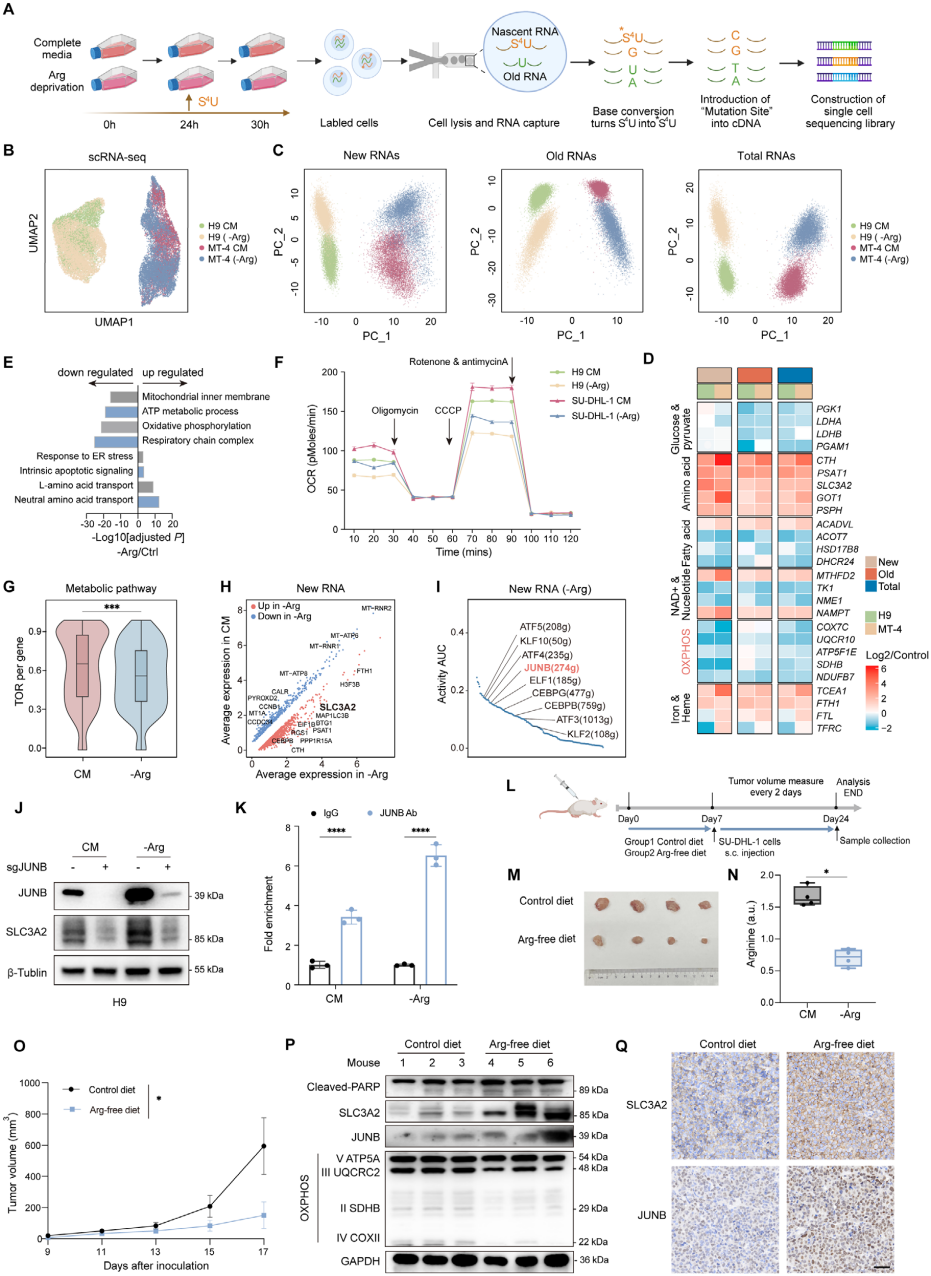
Single‐cell dynamic RNA sequencing reveals that arginine deficiency reduces OXPHOS and induces nascent RNA of SLC3A2 via JUNB. A) Schematic of single‐cell dynamic RNA sequencing. B) Uniform manifold approximation and projection (UMAP) plots of H9 and MT‐4 cells in complete medium (CM) or arginine deprivation (‐Arg) medium. C) Principal component analysis (PCA) showing the four groups (H9 and MT‐4 cells in complete medium or arginine deprivation medium) according to new, old, and total RNAs. D) Heatmap of a subset of metabolic DEGs in the arginine deprivation group compared to those in complete medium group (log2 fold change) according to new, old, and total RNAs. E) The enriched Gene Ontology (GO) pathway –log 10 (adjusted *p* values) of gene sets significantly changed in the arginine deprivation group versus complete medium group. F) Seahorse analysis of H9 and SU‐DHL‐1 cell oxygen consumption rates (OCRs) after exposure to complete or arginine‐depleted medium for 24 h. Time points of oligomycin (1 × 10^−6^ m), CCCP (1 × 10^−6^ m), and antimycin A (1 × 10^−6^ m) addition are indicated (*n* = 3). G) Violin plots of the turnover rate (TOR) of metabolic genes in complete medium and arginine deprivation groups. H) Volcano plot of differentially expressed new RNAs in H9 and MT‐4 cells in the complete medium or the arginine deprivation group. The top 20 of differentially expressed new RNAs are labeled. I) Activity AUC of pySCENIC‐predicted transcription factors (TFs) in new RNAs from the arginine deprivation group. Nine upregulated TFs potentially bind to the SLC3A2 promoter, and the total number of their target genes is labeled. J) Immunoblots of Cas9+ H9 cells expressing nontargeting control or independent sgRNAs targeting *JUNB* in complete or arginine‐depleted medium (*n* = 3). β‐Tubulin serves as a loading control. K) Nuclear extracts of H9 cells incubated in complete or arginine‐depleted medium were immunoprecipitated and analyzed using chromatin immunoprecipitation (ChIP) (*n* = 3). IgG serves as a control. L) Schematic representation of control versus arginine‐free diet intervention in a SU‐DHL‐1 xenograft mouse model. M) Photographs of tumors in the control diet and arginine‐free diet‐fed mice (*n* = 4). N) Liquid chromatography–mass spectrometry (LC‐MS/MS) detected the arginine levels in the tumors of control and arginine‐free diet‐fed mice (*n* = 4). O) Tumor growth curves of the control diet and arginine‐free diet‐fed mice (*n* = 4). P) Immunoblots of whole‐cell lysates from tumors of control and arginine‐free diet‐fed mice (*n* = 3). GAPDH serves as a loading control. Q) Representative Immunohistochemistry (IHC) staining showing JUNB and SLC3A2 expression in the tumors of the control and arginine‐free diet‐fed mice (*n* = 4). Scale bar, 50 × 10^−6^ m. For all panels, the data are presented as means ± SD. ^*^ *p* < 0.05; ^**^ *p* < 0.01; ^***^ *p* < 0.001; ^****^ *p* < 0.0001; ns, nonsignificant. For E, *p* values were generated using a permutation test. For H and G, *p* values were generated using the Wilcoxon‐rank sum test. For K, *p* values were generated using a two‐way ANOVA with multiple comparisons. For N and O, *p* values were generated using Student's two‐tailed unpaired *t*‐test.

Next, we investigated the mechanisms by which arginine promoted the growth of PTCL cells. Arginine availability forms a nexus for the synthesis of polyamines, creatine, and proline, which are essential for cell survival and proliferation.^[^ ^53^ ^]^ Key enzymes involved in the arginine catabolic pathway, including GAMT, AGMAT, ARG2, and OAT, were transcriptionally upregulated during AITL progression (Figure 3F). However, supplementation with ornithine, an upstream intermediate involved in proline synthesis; three major polyamine species (putrescine, spermine, and spermidine); and creatine only slightly rescued the growth of PTCL cells under arginine‐restricted conditions (Figure S5A, Supporting Information). This suggests that the effects of arginine restriction cannot be fully accounted for by disrupting arginine catabolism. Arginine directly impacts several metabolic pathways in immune and cancer cells.^[^ ^18^, ^47^ ^]^ To understand whether unmetabolized arginine contributed to the metabolic reprograming of PTCL cells by regulating metabolic gene expression, we used single‐cell dynamic RNA sequencing, a new technology developed by combining dynamic transcriptomes with high‐throughput single‐cell sequencing technology.^[^ ^54^, ^55^ ^]^ We revealed dynamic changes in PTCL cell transcripts by labeling the newly synthesized mRNA to distinguish between old and nascent RNAs after incubating H9 and MT‐4 cells with complete medium or arginine deprivation medium for 24 h (**Figure** **5**A).

Cells cultured in arginine‐restricted medium were markedly separated from control cells regarding new, old, and total RNAs in the PCA (Figure 5B,C). Single‐cell Metabolism (sc‐Metabolism) and hallmark analysis revealed that arginine restriction significantly altered several metabolic pathways, including those related to oxidative phosphorylation, amino acids, NAD+, nucleotides, and fatty acids (Figure S5B,C, Supporting Information). The differential expression of new, old, and total RNAs of the signature genes is shown in Figure 5D. Among these, OXPHOS was the most significantly downregulated among these metabolic pathways (Figure S5C,D, Supporting Information). Similarly, GO analysis showed that arginine restriction significantly downregulated oxidative phosphorylation and impaired mitochondrial respiratory function (Figure 5E), which is consistent with the reported effects of arginine on OXPHOS and mitochondrial activity in T cells.^[^ ^47^ ^]^ Arginine‐restricted PTCL cells exhibited increased mitochondrial reactive oxygen species levels and decreased ATP levels and oxygen consumption rates (OCRs) (Figure 5F, Figure S5E,F, Supporting Information), suggesting that arginine starvation resulted in PTCL bioenergetic stress. The turnover rate (TOR) reflects the degree of new RNA synthesis.^[^ ^55^ ^]^ The TOR of the metabolic pathways altered by arginine according to sc‐Metabolism analysis in the arginine deprivation group was significantly lower than that in the complete medium (Figure 5G), indicating that arginine restriction inhibited the production of new RNAs for critical genes in several metabolic pathways. These results suggest that arginine controls oncogenic metabolism reprogramming, especially enhancing OXPHOS at the transcriptional level by promoting new RNA synthesis, leading to PTCL growth.

Notably, GO analysis also revealed that arginine restriction induced amino acid transport activity (Figure 5E), corresponding with our in vitro results (Figure 4B). We predicted that PTCL cells would initiate the upregulation of SLC3A2 transcription in a cell‐autonomous manner to compensate for environmental arginine deficiency. As expected, nascent SLC3A2 RNA levels significantly increased in the arginine restriction group (Figure 5H), indicating that SLC3A2 was transcriptionally active. We then explored the mechanisms underlying the increased SLC3A2 transcription in PTCL. The Python Package Transcription Factor Regulatory Network analysis (pySCENIC) predicted 16 transcription factors that may bind to the SLC3A2 promoter region. Among them, nine exhibited upregulated regulatory activity under arginine deprivation (Figure 5I). We then used small interfering RNA (siRNA) to knock down these nine genes in PTCL cells. Surprisingly, *JUNB* knockdown significantly reduced *SLC3A2* mRNA levels (Figure S5G, Supporting Information). Immunoblotting confirmed that *JUNB* knockdown abrogated SLC3A2 protein expression (Figure S5H, Supporting Information). Indeed, arginine deprivation stimulated SLC3A2 expression in a JUNB‐dependent manner (Figure 5J and Figure S5I, Supporting Information). Chromatin immunoprecipitation (ChIP) analysis showed robust JUNB binding to the SLC3A2 promoter in PTCL cells, and this was substantially promoted by arginine deprivation (Figure 5K, and Figure S5J, Supporting Information). These results demonstrate the mechanism of JUNB hyperactivation‐driven SLC3A2 expression in PTCL cells, particularly under arginine stress.

Next, we sought to determine whether tumor cells relied on arginine in vivo. Mice bearing subcutaneous SU‐DHL‐1 xenograft tumors were fed an arginine‐free or control diet from 1 week before tumor inoculation until tumor collection (Figure 5L). The mice tolerated this treatment well (Figure S5K, Supporting Information), and tumoral arginine levels were significantly reduced (Figure 5N). Arginine deprivation significantly impaired tumor growth and cell proliferation, coinciding with an increase in cell death (Figure 5M–P). Moreover, the levels of the OXPHOS complex, which is located in the mitochondrial inner membrane, were reduced in arginine‐free tumors (Figure 5P), strengthening the notion that arginine positively regulates OXPHOS status in PTCL. Furthermore, we observed that JUNB–SLC3A2 signaling vigorously increased under arginine‐free conditions (Figure 5P,Q, and Figure S5L, Supporting Information), indicating that the JUNB–SLC3A2 axis governs intracellular arginine homeostasis in PTCL in a cell‐autonomous manner.

### 2.6 Quinacrine Transcriptionally Regulates SLC3A2 via SSRP1 Targeting in a JUNB‐Dependent Manner

Based on the previous results, we investigated the mechanism through which quinacrine regulates SLC3A2 expression. Quinacrine has been shown to target SSRP1, which is a subunit of the FACT complex.^[^ ^56^, ^57^ ^]^ FACT is a histone chaperone present in chromatin remodeling during DNA transcription, replication, and repair.^[^ ^58^ ^]^ We speculated that quinacrine transcriptionally regulates SLC3A2 by targeting SSRP1 in PTCL. We first evaluated SSRP1 expression in PTCL cell lines and found that SSRP1 protein levels were higher in PTCL cells than in normal T cells (Figure S6A, Supporting Information). We further evaluated the effect of quinacrine on SSRP1 expression and confirmed that quinacrine inhibited SSRP1 expression in a dose‐dependent manner (Figure S6B, Supporting Information).

**Figure 6:**
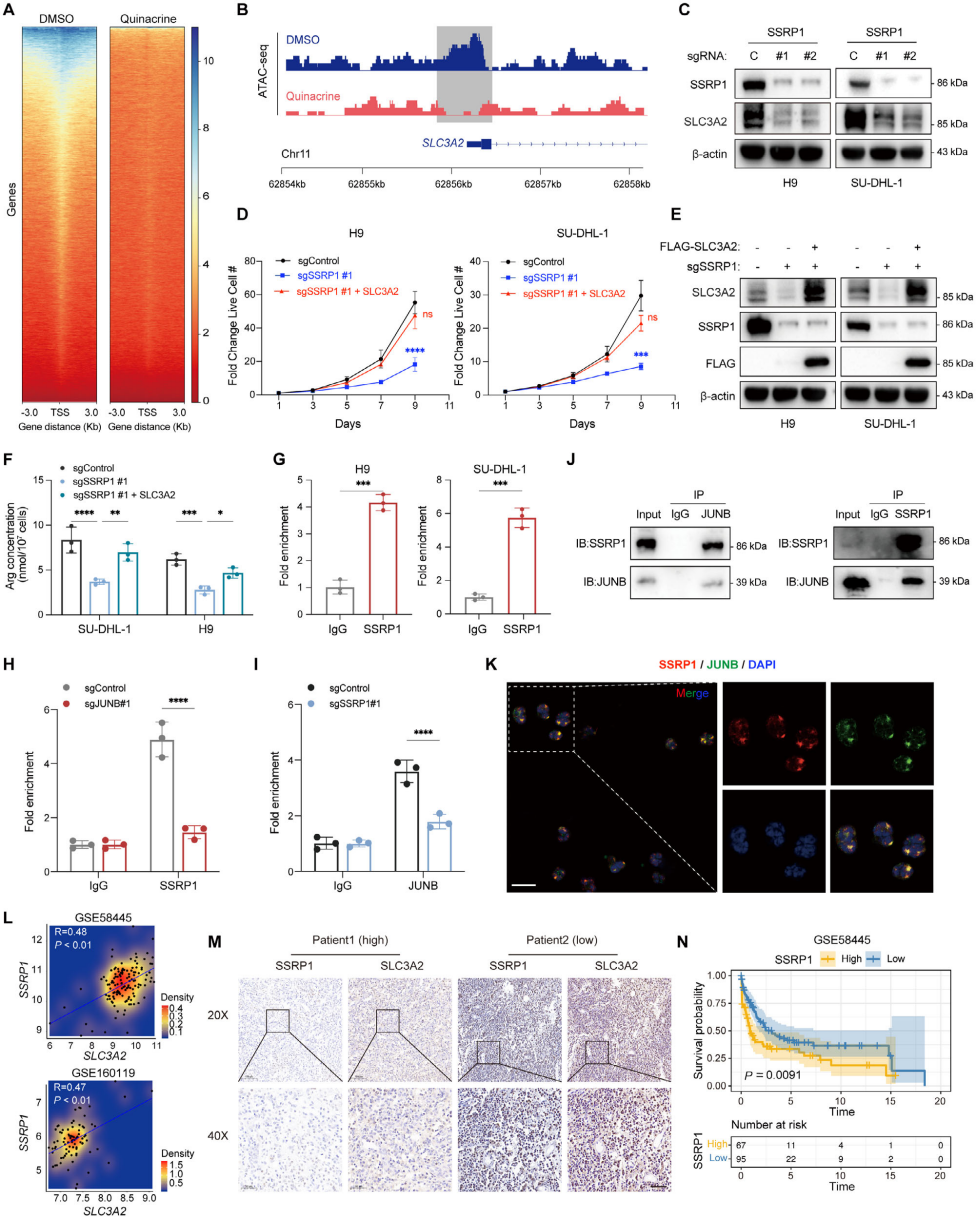
Quinacrine transcriptionally regulates SLC3A2 by targeting SSRP1 in a JUNB‐dependent manner A) ATAC‐seq heatmap comparing the landscapes of accessible chromatin in H9 cells treated with 2 × 10^−6^ m quinacrine or dimethyl sulfoxide (DMSO) for 48 h. B) Integrated Genomics Viewer screenshot depicting tracks from ATAC‐seq at the SLC3A2 locus. C) Immunoblot analysis of SLC3A2 expression in whole‐cell lysates from Cas9+ H9 and SU‐DHL‐1 cells expressing control or independent *SSRP1*‐targeting sgRNAs (*n* = 3). β‐actin serves as a loading control. D,E) Growth curves of Cas9+ H9 (left) and SU‐DHL‐1 (right) cells expressing nontargeting control or independent sgRNAs against *SSRP1* and transfected with flag‐SLC3A2 or a control vector (D). Immunoblots of indicated proteins are shown in E (*n* = 3). F) Arginine levels in Cas9+ H9 and SU‐DHL‐1 cells expressing nontargeting control or independent sgRNAs against *SSRP1*, transfected with flag‐SLC3A2 or the control vector (*n* = 3). β‐actin serves as a loading control. G) A chromatin immunoprecipitation (ChIP) assay was performed in H9 and SU‐DHL‐1 cells using anti‐SSRP1 antibodies, followed by RT‐qPCR. The fold change in the expression of ChIP‐enriched mRNAs relative to the input was calculated (*n* = 3). IgG serves as a control. H) A ChIP assay was performed on H9 cells expressing control or *JUNB* sgRNA using anti‐SSRP1 or anti‐IgG antibodies, followed by RT‐qPCR. The fold change in the expression of ChIP‐enriched mRNAs relative to the input was calculated (*n* = 3). IgG serves as a control. I) A ChIP assay was performed on H9 cells expressing control or *SSRP1* sgRNA using anti‐JUNB or anti‐IgG antibodies, followed by RT‐qPCR. The fold change in the expression of ChIP‐enriched mRNAs relative to the input was calculated (*n* = 3). IgG serves as a control. J) Immunoblots of SSRP1 and JUNB from anti‐JUNB immunoprecipitants (IPs) (left) and anti‐SSRP1 IPs (right) obtained from H9 cells (*n* = 3). K) Representative immunofluorescence staining of JUNB and SSRP1 in H9 cells (*n* = 3). Scale bar, 25 × 10^−6^ m. L) Scatter plot showing the correlation between SSRP1 and SLC3A2 expression in bulk RNA sequencing datasets (GSE160119 and GSE58445). M) Representative images showing the correlation between SSRP1 and SLC3A2 staining in human peripheral T‐cell lymphoma (PTCL) samples. Scale bars, 50 × 10^−6^ m. N) Kaplan–Meier curves of overall survival (OS) in PTCL patients with different SSRP1 expression levels in the GSE58445 dataset. For all panels, the data are presented as means ± SD. ^*^ *p* < 0.05; ^**^ *p* < 0.01; ^***^ *p* < 0.001; ^****^ *p* < 0.0001; ns, nonsignificant. For D and F, *p* values were generated using one‐way ANOVA with multiple comparisons. For G, *p* values were generated using Student's two‐tailed unpaired t‐test. For H and I, *p* values were generated using two‐way ANOVA with multiple comparisons. For L, *p* values were generated using Pearson's test. For N, *p* value was generated using the log‐rank test.

We investigated the differences in chromatin accessibility between PTCL cells treated with quinacrine and dimethyl sulfoxide (DMSO) using a transposase‐accessible chromatin assay with high throughput sequencing (ATAC‐seq). A remarkable decrease in signal intensity was observed over the accessible chromatin regions in quinacrine‐treated cells compared to control cells, indicating reduced transcriptional activity in the former (**Figure** **6**A). A focused analysis of the ATAC‐seq peaks at the SLC3A2 locus showed that the control cells had a peak position around the SLC3A2 transcription start site, representing an ‘‘open chromatin’’ configuration; no such peak was observed in the quinacrine‐treated group (Figure 6B). To further assess the role of SSRP1 in SLC3A2 expression, we used CRISPR editing, as confirmed by immunoblotting, to knockout SSRP1 in PTCL cells (H9 and SU‐DHL‐1). SSRP1 knockout blocked SLC3A2 mRNA and protein expression before inducing cell death (Figure 6C and Figure S6C, Supporting Information). We overexpressed SLC3A2 in SSRP1‐knockout cells (Figure 6E) and found that SLC3A2 cDNA reversed the inhibition of cell proliferation (Figure 6D) and restored arginine levels in cells expressing sgSSRP1 (Figure 6F). SSRP1 occupancy at the SLC3A2 locus was confirmed by ChIP followed by quantitative polymerase chain reaction (ChIP‐qPCR) (Figure 6G).

Given that the transcription factor JUNB plays a crucial role in SLC3A2 transcriptional regulation, especially under arginine‐free conditions, we assumed that SSRP1 may work cooperatively with JUNB to regulate SLC3A2 expression. To further test our hypothesis, we initially detected *SLC3A2* mRNA levels and observed that under SSRP1 knockout, JUNB knockout did not decrease *SLC3A2* expression. Similarly, under JUNB knockout, SSRP1 knockout did not decrease *SLC3A2* expression (Figure S6D, Supporting Information). Subsequently, we performed ChIP‐qPCR and discovered that the interaction between SSRP1 and the SLC3A2 promoter was weakened by JUNB knockout. Similarly, the interaction of JUNB with the SLC3A2 promoter was weakened by SSRP1 knockout (Figure 6H,I, Figure S6E,F, Supporting Information). The interaction between SSRP1 and JUNB in PTCL cells was confirmed by co‐immunoprecipitation (co‐IP) and immunofluorescence confocal microscopy (Figure 6J,K, Figure S6G,H, Supporting Information). Additionally, immunoblotting confirmed that quinacrine did not affect JUNB expression (Figure S6I, Supporting Information). CBL0137, a lead compound modeled on quinacrine, has been investigated as a FACT inhibitor in solid tumors.^[^ ^56^, ^59^, ^60^, ^61^ ^]^ As expected, the pharmacological targeting of FACT with CBL0137 produced a similar effect in PTCL (Figure S6J–L, Supporting Information). Collectively, these results suggest that SSRP1 cooperates with JUNB to regulate SLC3A2 expression at the transcriptional level and highlight that quinacrine and CBL0137, as SSRP1 inhibitors, may represent an intriguing therapeutic drug for PTCL.

Next, we investigated the clinical and pathological relevance of SSRP1 in PTCL. The analysis of GSE160119 and Group1 (scRNA‐seq) revealed that *SSRP1* mRNA expression was significantly higher in PTCL tumor cells and increased during progression (Figure S6M,N, Supporting Information). A significant positive correlation was observed between *SSRP1* and *SLC3A2* mRNA expression in both GSE58445 (*p* < 0.01, *R* = 0.48) and GSE160119 datasets (*p* < 0.01, *R* = 0.47) (Figure 6L). IHC analysis of primary AITL samples from our center showed that SSRP1 protein levels were higher in AITL tissues than in normal tissues and were significantly correlated with SLC3A2 protein levels (*p* < 0.0001, *R* = 0.732) (Figure 6M and Figure S6O–Q, Supporting Information). Nevertheless, Kaplan–Meier survival analysis of both the GSE58445 dataset and our AITL cohort showed that high SSRP1 expression was significantly associated with poor OS in patients with PTCL (Figure 6N and Figure S6R, Supporting Information).

### 2.7 Combination Epigenetic Therapy Exerts a Synergistic Antitumor Effect on PTCL

**Figure 7:**
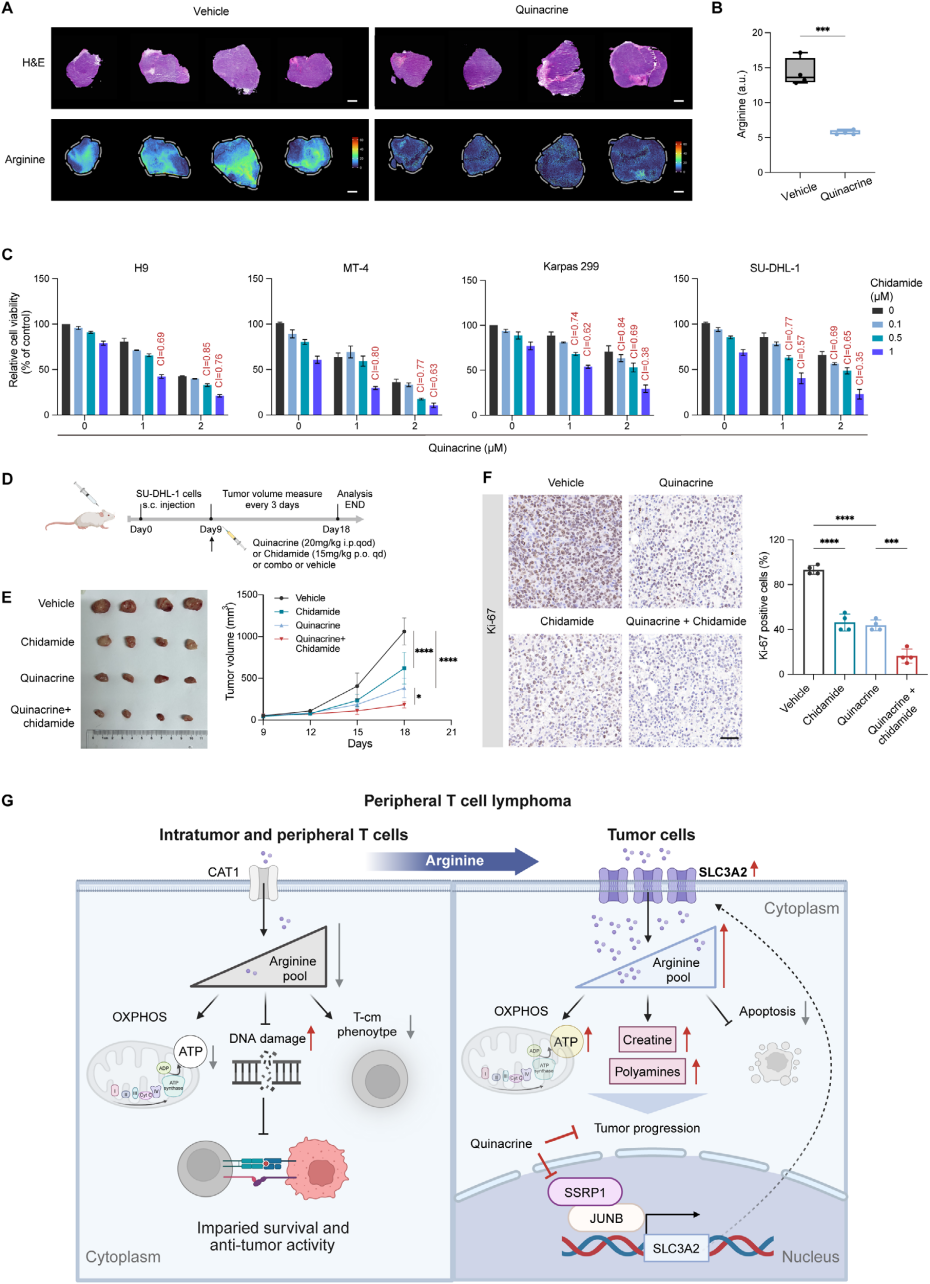
Combination epigenetic therapy exerts a synergistic antitumor effect on peripheral T‐cell lymphoma (PTCL). A,B) Matrix‐assisted laser desorption ionization mass spectrometry imaging (MALDI‐MSI) ion images (A) and quantified relative changes (B) for SU‐DHL‐1 xenografts treated with quinacrine or vehicle (*n* = 4). Scale bar, 2 mm. C) CCK8 analysis of the relative proliferation activity of the PTCL cell lines treated with quinacrine in combination with chidamide at different concentrations for 48 h (*n* = 3). The combination index (CI) is red‐coded. D) Schematic representation of a SU‐DHL‐1 xenograft mouse model treated with vehicle, quinacrine, chidamide, or quinacrine plus chidamide combination therapy in vivo. E) Tumor volume (left) and tumor growth curve (right) in the SU‐DHL‐1 xenograft mouse model treated with vehicle only (black), chidamide (green), quinacrine (blue), and quinacrine in combination with chidamide (red) (*n* = 4). F) Representative images (left) of immunohistochemistry (IHC) staining showing Ki‐67 expression in tumor samples from the four groups (vehicle, chidamide, quinacrine, and quinacrine plus chidamide groups) and the corresponding quantification (right) (*n* = 4). Scale bar, 50 × 10^−6^ m. G) Schematic model. In PTCL, SLC3A2‐mediated excessive arginine uptake by tumor cells produces arginine deficiency in the TME and circulation, thus impairing CD8^+^ T‐cell survival and function, contributing to the immune escape of PTCL cells. An abundant arginine pool in tumor cells upregulates arginine catabolic activity; induces global metabolic changes, including enhanced oxidative phosphorylation; and inhibits apoptosis, thereby fueling tumor progression. SSRP1 upregulates SLC3A2 as a co‐transcription factor with JUNB. Quinacrine disrupts SLC3A2‐mediated arginine transport by targeting SSRP1 and represents a promising therapeutic strategy for PTCL. For all panels, the data are presented as means ± SD. ^*^ *p* < 0.05; ^**^ *p* < 0.01; ^***^ *p* < 0.001; ^****^ *p* < 0.0001; ns, nonsignificant. For B, *p* values were generated using Student's two‐tailed unpaired t‐test. For E and F, *p* values were generated using one‐way ANOVA with multiple comparisons.

Tumors are intrinsically heterogeneous, requiring tumor cells to constantly rewire their metabolic pathways. This, coupled with the high degree of metabolic flexibility exhibited by many tumors, represents a notable obstacle in cancer therapy.^[^ ^62^ ^]^ To further validate the metabolic effects of quinacrine in situ, we used matrix‐assisted laser desorption ionization mass spectrometry imaging (MALDI‐MSI). Mass spectrometry imaging visualized arginine depletion throughout the tumor tissue in SU‐DHL‐1 tumor‐bearing mice treated with quinacrine (**Figure** **7**A,B), supporting the validity of the observed effects of quinacrine on intratumoral arginine in vivo. IHC analysis demonstrated that quinacrine downregulated the expression of SLC3A2 in vivo (Figure S7A,B, Supporting Information).

PTCL represents a group of prototypical epigenetic malignancies with an invariably poor prognosis.^[^ ^63^ ^]^ Epigenetic modifying agents, including histone deacetylase (HDAC) inhibitors such as chidamide, have demonstrated broad clinical efficacy and durability in PTCL and are being clinically developed as combination strategies in both relapse and frontline settings.^[^ ^64^, ^65^, ^66^ ^]^ Given the effect of quinacrine on FACT inhibition, we hypothesized that quinacrine may further potentiate the effect of HDAC inhibitors. As expected, the combination of quinacrine/CBL0137 and chidamide significantly impaired PTCL growth with combination indices, indicating a potent synergy of <1 in all tested cell lines (Figure 7C and Figure S7C, Supporting Information). Combination epigenetic therapy also exerted significantly enhanced antitumor effects compared to vehicle or single‐agent treatments without a noticeable body weight impact in vivo (Figure 7D–F, Figure S7D,E, Supporting Information). Overall, these findings indicated that quinacrine and the HDAC inhibitor chidamide have a synergistic antitumor effect on PTCL, suggesting a novel therapeutic strategy for treating this genetically heterogeneous disease.

## 3 Discussion

In this study, we identified quinacrine as an effective arginine transport inhibitor for the treatment of PTCL based on HTS. We observed aberrant arginine metabolic reprogramming in patients with PTCL, characterized by high expression of the arginine transporter SLC3A2. Increased arginine uptake via SLC3A2 promotes PTCL cell proliferation and impairs T‐cell antitumor immunity in the TME. The JUNB–SLC3A2 axis governs intracellular arginine homeostasis, and arginine directly controls global metabolic fitness in PTCL. Notably, the FACT complex subunit SSRP1 cooperates with JUNB to regulate SLC3A2 expression at the transcriptional level. Quinacrine disrupts SLC3A2‐mediated arginine transport by targeting SSRP1. Therefore, quinacrine treatment represents a promising therapeutic strategy for the treatment of PTCL.

Quinacrine is a 9‐aminoacridine derivative that has been approved for the treatment of malaria and cutaneous lupus erythematosus; in recent years, it has been identified as a potential antitumor agent and established as an effective drug for the treatment of hematologic malignancies, including acute myeloid leukemia and T‐cell acute lymphoblastic leukemia.^[^ ^37^, ^39^, ^40^, ^41^ ^]^ Nevertheless, quinacrine's antitumor effect mechanisms are not well understood. Quinacrine has been shown to disrupt DNA damage repair, target arachidonic pathways, induce p53, and inhibit FACT complex and NF‐κB signaling.^[^ ^38^, ^57^ ^]^ Here, we demonstrate for the first time that quinacrine exhibits excellent anti‐PTCL activity both in vitro and in vivo. Surprisingly, our findings show that quinacrine plays a unique regulatory role in the intracellular arginine PTCL pool.

Alterations in the arginine‐synthesizing urea cycle are common in various cancer types. Previous studies have demonstrated that the suppression of the urea cycle induces reliance on exogenous arginine sources and is associated with poor survival in various cancers.^[^ ^19^, ^67^, ^68^ ^]^ We also noted the silencing of the key arginine synthesis enzyme ASS1 in patients with PTCL, indicating that PTCL exhibits arginine auxotrophy. Arginine is involved in multiple downstream cellular metabolic pathways. Moreover, we found that the enzymes ARG2, OAT, GAMT, and AGMAT, which convert arginine into ornithine, proline, spermidine, and creatine, respectively, were upregulated during AITL progression. This observation is analogous to that in T cells, where arginine is metabolized upon activation.^[^ ^47^ ^]^ However, in contrast to the reduced arginine levels resulting from rapid catabolism in T cells, we observed increased arginine levels in PTCL cell lines and decreased arginine levels in the plasma of patients with PTCL, indicating that unmetabolized arginine may induce cell‐intrinsic alterations to promote PTCL progression.

Moreover, arginine auxotrophy makes cells dependent on an extracellular arginine supply to sustain high arginine levels. Arginine can be transported across membranes by SLC family proteins.^[^ ^27^ ^]^ In T cells, hepatocellular carcinoma cells, and chronic lymphocytic leukemia cells, SLC7A1 plays a dominant role in arginine transport.^[^ ^19^, ^28^, ^69^ ^]^ Interestingly, we observed that SLC3A2 was highly expressed in patients with PTCL, and this increased expression was associated with a significantly shorter OS in patients with AITL, though further validation is needed to determine the clinical relevance of SLC3A2 expression in other PTCL subtypes. Moreover, SLC3A2, rather than SLC7A1, is responsible for arginine uptake by PTCL.

Increasing arginine uptake via SLC3A2 enhances the proliferative and anti‐apoptotic capabilities of PTCL cells both in vivo and in vitro. Notably, SLC3A2 was highly expressed and predicted to be associated with a poor prognosis in patients with PTCL. To the best of our knowledge, no selective SLC3A2 inhibitors currently exist, and their future development is highly warranted. We demonstrated that quinacrine treatment downregulated the intracellular arginine pool by targeting SLC3A2 and selectively inhibiting tumor growth. Considering that the safety and tolerability of quinacrine have been identified in other hematological malignancies,^[^ ^39^, ^40^, ^41^ ^]^ quinacrine treatment can be quickly and feasibly translated into clinical practice to improve patient survival.

The nutritional and energetic interplay between tumor cells and intratumoral immune cells leads to metabolic competition.^[^ ^70^ ^]^ Arginine concentration plays a critical role in the survival capacity and antitumor immunity of cytotoxic CD8^+^ T cells.^[^ ^47^, ^49^ ^]^ Our study illustrates the competition for arginine between SLC3A2‐proficient PTCL cells and CD8^+^ T cells. Excessive SLC3A2‐mediated arginine uptake by PTCL cells produces arginine deficiency in the TME and circulation, thus impairing cytotoxic CD8^+^ T‐cell survival and function and contributing to immune escape by PTCL cells.

Arginine auxotrophy has led to the development of circulating arginine‐degrading enzymes and dietary arginine restriction as a therapeutic strategy.^[^ ^71^, ^72^, ^73^, ^74^ ^]^ However, the clinical benefits related to cancer progression and patient survival have been minimal, at least partly because of inhibitory T‐cell activation and expansion.^[^ ^71^, ^74^ ^]^ Notably, SLC3A2 is not responsible for arginine uptake in T cells; accordingly, we discovered that quinacrine selectively downregulated arginine levels in tumor cells, highlighting that quinacrine as an attractive therapeutic option that may avoid the undesirable side effects of T‐cell inhibition. We predict that immunotherapy with checkpoint inhibitors may be augmented by quinacrine signaling in PTCL.

How does arginine promote PTCL cell outgrowth? Arginine directly affects metabolism in immune and cancer cells,^[^ ^18^, ^47^ ^]^ which is supported by our observations. In T cells, arginine regulates several metabolic pathways and enhances OXPHOS.^[^ ^47^ ^]^ Arginine starvation enhanced OXPHOS and serine synthesis in leiomyosarcoma and melanoma cell lines.^[^ ^75^ ^]^ Conversely, in ASS1‐negative breast cancer cells and hepatocellular carcinoma cells, arginine deprivation reduced OXPHOS, permitting mitochondrial dysfunction.^[^ ^19^, ^20^ ^]^ Interestingly, in hepatocellular carcinoma cells, arginine binds to RBM39, a transcription cofactor that regulates metabolic gene expression at the transcriptional level.^[^ ^18^ ^]^ Similarly, we found that arginine also controls oncogenic metabolism reprogramming, especially by enhancing OXPHOS at the transcriptional level and promoting the new RNA synthesis of metabolic genes altered by arginine restriction, leading to PTCL growth. However, the transcription factors through which arginine induces broad metabolic changes in PTCL remain elusive. Further studies using a proteome‐wide analysis of metabolite–protein interactions may be necessary to uncover the underlying mechanisms.

How do PTCL cells maintain upregulated SLC3A2 expression, even under arginine restriction conditions? In our study, we found that the transcription factor JUNB, which is critical for T‐cell development,^[^ ^76^, ^77^ ^]^ transcriptionally regulates SLC3A2 expression under arginine‐free conditions, where the JUNB–SLC3A2 axis governs intracellular arginine homeostasis in PTCL in a cell‐autonomous manner. Furthermore, we confirmed that SSRP1 is a co‐transcription factor involved in the JUNB‐dependent regulation of SLC3A2. As the SSRP1 inhibitor quinacrine did not regulate JUNB, we concluded that SSRP1 functions in conjunction with JUNB to regulate SLC3A2 expression at the transcriptional level.

Previous studies demonstrated that quinacrine inhibits SSRP1 expression.^[^ ^38^, ^56^, ^57^ ^]^ CBL0137, a FACT complex inhibitor that was modeled on quinacrine, is also undergoing several clinical trials for hematological malignancies and solid tumors (NCT04870944, NCT01905228, NCT02931110, and NCT03727789). In this study, we demonstrated that quinacrine disrupts SLC3A2 transcription by targeting SSRP1, resulting in decreased arginine levels and the inhibition of PTCL cell growth. Notably, the combination of quinacrine or CBL0137 with HDAC inhibitors has demonstrated synergistic effects in multiple cancer types.^[^ ^40^, ^56^, ^78^ ^]^ In our study, quinacrine also exhibited a therapeutic synergy with HDAC inhibitors in PTCL both in vitro and in vivo, suggesting that this combination epigenetic therapy may represent a favorable therapeutic strategy for PTCL. The mechanism of interaction between quinacrine and HDAC inhibitors in PTCL remains elusive. A possible explanation for these synergistic effects is that the FACT complex, known to maintain nucleosome integrity through SSRP1 interactions with H3/H4 tetramers during transcription, may colocalize at the same genomic regions as specific histone modifications in PTCL. Further studies are required to clarify this mechanism.

## 4 Conclusion

Our findings demonstrate that quinacrine plays a crucial role in regulating the intracellular arginine pool by transcriptionally downregulating the arginine transporter SLC3A2, resulting in the inhibition of PTCL cell growth. Our work provides a rationale for initiating clinical studies on SSRP1 inhibitors quinacrine/CBL0137 in PTCL and outlines a new combination epigenetic therapy that shows promise for the treatment of genetically heterogeneous PTCL.

## 5 Experimental Section

### Cell Culture, Antibodies, and General Reagents

Human PTCL cell lines Hut78 and H9 (cutaneous T‐cell lymphoma cell lines), MT‐4 (adult T‐cell leukemia‐lymphoma cell line), and SU‐DHL‐1 (ALCL) were purchased from the BeNa Culture Collection. The human PTCL cell line Karpas 299 (ALCL) was purchased from Cobioer. All PTCL cell lines were cultured in RPMI‐1640 (Gibco, Cat# C11875500BT) supplemented with 10% standard fetal bovine serum (FBS) (VivaCell, Cat# C04001‐500) and 1% penicillin–streptomycin (Beyotime, Cat# C0222) in a humidified incubator at 37 °C with 5% CO_2_. 293T cells were obtained from the American Type Culture Collection and grown in Dulbecco's Modified Eagles Medium (Gibco, Cat# C11995500BT) supplemented with 10% FBS. All cell lines were authenticated by short tandem repeat analysis and were frequently checked for morphological features. They were routinely tested and certified as mycoplasma‐free using a MycoBlue Mycoplasma Detector (Vazyme, Cat# D101‐01).

PBMCs from healthy donors were isolated using lymphoprep density gradient medium (TBD Science, Cat# LTS1077), and CD8^+^ T cells were enriched with magnetic microbeads (Miltenyi Biotec, Cat# 130‐045‐201). Freshly purified human T cells were activated with the corresponding CD3/CD28 T‐cell activator (STEMCELL Technologies, Cat# 10971) and cultured in serum‐free hematopoietic cell medium (Lonza, Cat# 02–053Q) supplemented with 10% FBS and interleukin‐2 (10 ng/mL).

For experiments involving arginine deprivation, cells cultured in RPMI‐1640 with 10% regular FBS (complete medium) were washed three times with PBS and then resuspended in arginine‐free RPMI‐1640 (Pricella, Cat# DM150110) supplemented with 10% dialyzed FBS (Thermo Fisher Cat # 26400044). l‐Arginine was added to the culture media at the indicated concentrations, typically 2 × 10^−3^ m, and the cells were grown for the indicated times. Reagents and antibodies used in this study are presented in Data S1 (Supporting Information).

### Mice

The xenograft tumor experiments and procedures were reviewed and approved by the Ethics Committee of Nanjing Agricultural University (Permit No. PZW2024024). Four‐ to six‐week‐old female NCG (NOD/ShiLtJGpt‐Prkdcem26Cd52Il2rgem26Cd22/Gpt) mice were purchased from Gempharmatech Company (Shanghai, China). All mice were housed under specific pathogen‐free conditions and handled according to the Jackson Laboratory guidelines at the animal facility of the Animal Experimentation Center, Nanjing Agricultural University.

### Human Specimens

This study was reviewed and approved by the Ethics Committee of Nanjing Medical University (approval no. 2024‐SRFA‐176), and written informed consent was obtained from all participants involved in this study. Human lymphoid tissues were obtained from patients with PTCL who underwent ultrasound‐guided needle biopsies at the First Affiliated Hospital of Nanjing Medical University between 2018 and 2023. After obtaining written informed consent, fresh peripheral blood samples were obtained from patients with PTCL and healthy volunteers. Histological diagnoses were established according to the fourth edition of the World Health Organization classification of hematolymphoid tumors and were reviewed by two independent pathologists. In total, 80 samples from patients with PTCL (36 primary tumor samples and 44 peripheral blood samples) and five samples from healthy donors (peripheral blood samples) were included in this study. Eight AITL tumor tissue samples were used for scRNA‐seq (Group1). For validation, three AITL tumor tissue samples, four peripheral blood samples from patients with AITL, and five peripheral blood samples from healthy volunteers were included in scRNA‐seq Group 2 (Table S2, Supporting Information). All the patients received a CHOP/CHOP‐like regimen. The baseline demographics and clinical characteristics of patients with PTCL were assessed from patient records. The Ethics Committee of Nanjing Medical University approved all experiments involving human tissue and blood samples that were used for IHC staining and biochemical assessments of arginine levels reported in this study. Tissue microarrays containing human primary PTCL and RLH tissues were purchased from Outdo Biotech (Shanghai, China). Patient clinical, demographic, and pathological information are shown in Table S3 (Supporting Information).

### Drug Screening

A custom‐curated metabolic inhibitor drug library was purchased from Selleck and contained 203 Food and Drug Administration‐approved drugs. Multiple inhibitors of well‐explored metabolic targets such as fatty acid synthase, pyruvate dehydrogenase kinase, and dihydrofolate reductase were included while simultaneously considering mechanistic diversity and targeting distinct metabolic pathways (Table S1, Supporting Information). Screening was performed using the HTS platform of China Pharmaceutical University. Four PTCL cell lines (H9, Hut78, Karpas 299, and MT‐4) were used as preclinical models to assess drug efficacy against PTCL. The seeded cells were plated and incubated with each tested compound at a final concentration of 50 × 10^−6^ m for 72 h. Cells incubated with doxorubicin for 24 h only served as a positive control. AlamarBlue cell viability reagent (10%) (Thermo Fisher Scientific, Cat# DAL1025) was added to each cell, followed by 4 h incubation at 37 °C. The reaction was terminated by adding SDS solution (50 µL of 3% SDS). Fluorescence intensity was detected using a microplate reader (Figure 1A). Drugs were ranked based on their cell inhibition rates. Hits were defined as drugs that displayed >50% inhibition rate values for cell lines. Three hits were identified in all the four cell lines (Figure 1D).

### Cell Viability Assay and Growth Curve Analysis

Cells subjected to the indicated treatments were seeded in a 96‐well plate (1 × 10^4^ cells with 100 µL culture medium per well) for the cell viability assay. At each time point, 10 µL of Cell Counting Kit‐8 (CCK8; Dojindo, Cat# CK‐04) reagent was added to each well. After 2 h of incubation at 37 °C protected from light, the optical density value at 450 nm wavelength was subsequently measured using a microplate reader (ELx800, Bio‐Tek, USA).

For growth curve analysis, the cells were normalized to the same starting concentration. The cell numbers were quantified using a CellTiter‐Glo assay at the indicated time points.

### Cell Apoptosis Analysis

Cells undergoing the indicated treatments were harvested, washed once with cold PBS, and stained with 5 µL of Annexin V‐FITC/APC antibody and propidium iodide (PI) for 5 min at room temperature (RT). Cells were washed twice, resuspended in FACS buffer (3% FBS in PBS), and immediately processed. Flow cytometric data were acquired using a Beckman Coulter instrument, and analyses were performed using FlowJo V10.

### Cell Cycle Analysis

A total of 5 × 10^6^ cells were seeded in a T25 flask containing 10 mL complete, or arginine‐free medium. Two days after seeding, cells were harvested, fixed using ice‐cold 70% ethanol, and stored at −20 °C overnight. Following centrifugation (500 × *g*, 5min) and ethanol removal, the cells were washed twice in PBS and then resuspended in 500 µL PI staining solution (Liankebio, Cat# CCS012). The cells were incubated at RT in the dark for 30 min prior to flow cytometry.

### Metabolite Profiling and Statistical Analysis

Untargeted LC‐MS/MS and targeted ultra‐high‐performance LC‐MS (UHPLC) were performed by Biotree (Shanghai, China).

For untargeted metabolite profiling of H9 cells, 5 × 10^6^ cells were seeded into a T25 flask and incubated with DMSO or quinacrine (2 × 10^−6^ m) for 48 h. Cells were pelleted, resuspended with pre‐chilled 80% methanol by vortexing for 30 s, and incubated in liquid nitrogen for 1 min. After thawing at RT, the samples were sonicated for 10 min at 4 °C in a water bath and incubated for 1 h at −40 °C to precipitate proteins. The samples were then centrifuged at 12 000 rpm for 15 min at 4 °C. The supernatant was then transferred to a fresh glass vial for further analysis. A quality control sample was prepared by mixing equal aliquots of the supernatants from the samples.

Untargeted metabolite profiling was performed using a UHPLC system (Vanquish, Thermo Fisher Scientific) with a Waters ACQUITY UPLC BEH Amide column (2.1 mm × 50 mm, 1.7 × 10^−6^ m) coupled to an Orbitrap Exploris 120 mass spectrometer (Orbitrap MS, Thermo). MS/MS spectra were acquired in information‐dependent acquisition mode under the control of the acquisition software (Xcalibur, Thermo). The acquisition software continuously evaluated full‐scan MS spectra in this mode. The electrospray ionization (ESI) source conditions were set as follows: sheath gas flow rate, 50 Arb; Aux gas flow rate, 15 Arb; capillary temperature, 320 °C; full MS resolution, 60,000; MS/MS resolution, 15 000; collision energy, SNCE 20/30/40; and spray voltage, 3.8 kV (positive) or −3.4 kV (negative).

For statistical analysis of the untargeted metabolomics data, the raw data were converted to mzXML format using ProteoWizard and processed with an in‐house program developed using R and based on XCMS for peak detection, extraction, alignment, and integration. An in‐house MS2 database (BiotreeDB) was used for metabolite annotation. The cutoff for annotation was set at 0.3. Significantly deregulated metabolites were determined and visualized using the volcano plot function. Thresholds were set to variable importance in the projection (VIP) > 1 and *p <* 0.05. Commercial databases, including KEGG (http://www.genome.jp/kegg/) and MetaboAnalyst (http://www.metaboanalyst.ca/), were used for pathway enrichment analysis.

For amino acid profiling of MT‐4 cells transfected with control versus SLC3A2‐OE vectors, 0.5–1 × 10^7^ control or SLC3A2‐OE cells were pelleted and resuspended in fresh medium for 2 h prior to intracellular metabolite extraction. UHPLC was performed using an Agilent 1290 Infinity II Series UHPLC System (Agilent Technologies). An Agilent 6460 triple quadrupole mass spectrometer (Agilent Technologies) equipped with an AJS‐ESI interface was used for MS analysis in multiple reaction monitoring (MRM) mode. The Agilent MassHunter Work Station software (B.08.00, Agilent Technologies) was used for MRM data acquisition and processing.

### Arginine Measurement

Arginine levels in plasma, culture media, and cell lines were measured using an Arginine assay kit (Abcam, Cat# ab252892) according to the manufacturer's instructions. The arginine concentration was normalized to the number of cells or the protein concentration. To detect arginine in mouse xenograft tumors, samples were analyzed using LC‐MS/MS‐based metabolite analysis, as described above.

### Single Cell Collection, Sequencing, and Data Analysis

i) Single Cell Collection

For fresh lymph nodes, samples were stored in sCelLive Tissue Preservation Solution (Singleron Bio Com, Nanjing, China) on ice after the biopsy within 30 min, washed with Hanks’ balanced salt solution three times, and then digested with 2 mL sCelLive Tissue Dissociation Solution (Singleron) using a Singleron PythoN Automated Tissue Dissociation System (Singleron) at 37 °C for 15 min. The mixture was then centrifuged at 300 × *g* and 4 °C for 5 min to remove supernatant and gently suspended in PBS. For fresh peripheral blood, the GEXSCOPE red blood cell lysis buffer (Singleron, 2 mL) was added to samples and incubated at 25 °C for 10 min. Cells were isolated and suspended in PBS. For PTCL cell lines, cells subjected to the indicated treatments were harvested and resuspended in PBS to obtain single‐cell suspensions. Cells with a viability higher than 85% were subjected to scRNA‐seq.

ii) Library Preparation and scRNA‐seq

Single‐cell suspensions (2 × 10^5^ cells mL^−1^) in PBS were loaded onto a microwell chip using the Singleron Matrix Single Cell Processing System. Barcoding beads were subsequently collected from the microwell chip, followed by reverse transcription of the mRNA captured by the barcoding beads, cDNA synthesis, and PCR amplification. The amplified cDNA was fragmented and ligated using sequencing adapters. The scRNA‐seq libraries were constructed according to the protocol of the GEXSCOPE Single‐Cell RNA Library Kit (Singleron).^[^ ^79^ ^]^ Individual libraries were diluted to 4 × 10^−9^ m, pooled, and sequenced on an Illumina NovaSeq 6000 platform with 150 bp paired‐end reads.

iii) scRNA‐seq Quantification and Data Analysis

Raw reads from scRNA‐seq were processed to generate gene expression matrices using the CeleScope (https://github.com/singleron‐RD/CeleScope) v1.9.0 pipeline. Seurat v3.1.2 was used for quality control, dimensionality reduction, and clustering using Python 3.7. For each sample dataset, the expression matrix was filtered using the following criteria: 1) cells with a gene count less than 200 or with the top 2% gene count were excluded; 2) cells with top 2% unique molecular identifier (UMI) count were excluded; 3) cells with mitochondrial content > 20% were excluded; and 4) genes expressed in fewer than five cells were excluded. After filtering, qualified cells were retained for the downstream analyses. The raw count matrix was normalized to total counts per cell and logarithmically transformed into a normalized data matrix. The top 2000 variable genes were selected by setting flavor = “Seurat.” PCA was performed on the scaled variable gene matrix, and the top 20 principal components were used for clustering and dimensional reduction. Cell clusters were visualized using uniform manifold approximation and projection (UMAP).

To identify the DEGs between different samples or consecutive clusters, the Seurat FindMarkers function and selected genes expressed in more than 10% of the cells were used in a cluster with an average log(fold change) value greater than 0.25. For the cell‐type annotation of each cluster, the expression of canonical markers found in the DEGs was combined with information from the literature. To obtain a high‐resolution map of T cells, cells from a specific cluster were extracted and reclustered for a more detailed analysis, following the procedures described above. Tfh cell subsets were defined as described in our previous study.^[^ ^45^ ^]^ Briefly, T follicular helper (Tfh) cells were identified by the expression of seven marker genes (*PDCD1, ICOS, CXCR5, BCL6, MME, CD4*, and *CD40L*), and the Tfh score was constructed accordingly. The T cell clusters with the top three Tfh scores were identified as Tfh tumor cells. To investigate the potential functions of DEGs or specifically expressed genes, GO and KEGG analyses were performed using the “clusterProfiler” R package (v4.0.0). For gene set variation pathway enrichment analysis, the average gene expression in each cell type was provided as input data using the GSVA package. Gene set scoring was performed using the R package UCell (v2.2.0). UCell scores were based on the Mann–Whitney U test by ranking query genes according to their expression levels in individual cells. KEGG (hsa00330 and hsa00220) and GO (0006525) pathways were used as functional gene sets for UCell scoring.

### Single‐Cell Dynamic RNA Sequencing

For single‐cell dynamic RNA sequencing of H9 and MT‐4, 2 × 10^6^ cells were seeded into a T25 flask with 10 mL of complete media or arginine free media for 24 h. Then, for metabolic labeling in vitro, according to the protocol of the DynaSCOPE Single Cell Dynamic RNA Library Kits (Singleron), Labeling reagent (Singleron) was thawed at RT, prepared labeling culture medium, added labeling reagent to medium at 1:100 ratio and mixed well. The prepared cells were then transferred to the labeling culture medium and incubated away from light. After incubation for 6 h, cells were pelleted and resuspended in an appropriate volume of PBS. After library preparation, scRNA‐seq, primary analysis of raw read data, quality control, dimension‐reduction, and clustering as described above, matrices of new, old, and total transcripts were used in further analysis.

DEG analysis, pathway enrichment analysis, and UCell gene set scoring were performed as described above. PySCENIC analysis was performed using pySCENIC (v0.11.0) with the scRNA expression matrix and transcription factors from the AnimalTFDB. GRNBoost2 was used to predict a regulatory network based on the coexpression of regulators and targets. CisTarget was then used to exclude indirect targets and to search for transcription factor‐binding motifs. Subsequently, AUCell was used to quantify the regulon activity for every cell. Cluster‐specific TF regulons were identified using regulon‐specificity scores. The scMetabolism (v0.2.1) R package, designed to quantify single‐cell metabolic activity, was used for the analysis. KEGG metabolic pathways were determined, and metabolic pathway enrichment scores were calculated using VISION. The VlnPlot function in Seurat and a heatmap were used to visualize the scores of specific pathways. The metabolic pathway TOR, which was significantly altered by arginine restriction, was calculated as the proportion of newly synthesized transcripts among whole transcripts based on UMI counts in each gene or cell. The KEGG pathways hsa00190, hsa00230, hsa00240, hsa00030, hsa00010, hsa00100, and hsa00260 were included in the TOR calculations. A violin plot with a statistical test showed the TOR.

### Bulk RNA Dataset Analysis

PTCL datasets were downloaded from the Gene Expression Omnibus (GEO) database with accession IDs GSE160119 and GSE58445. DEG analysis of two conditions/groups (two biological replicates per condition) was performed in R using the “limma” (v3.50.1) package. GO and KEGG enrichment analyses were performed using the Cluster Profiler (v4.0.2) R package.

### Western Blot

Immunoblotting was performed as previously described.^[^ ^45^ ^]^ In brief, whole‐cell lysates prepared by boiling cells in 1× Laemmli buffer were separated by SDS‐PAGE electrophoresis, transferred onto polyvinylidene fluoride membranes, blocked with 5% milk in TBST buffer, and then probed with relevant primary antibodies at 4 °C overnight, followed by secondary antibody incubation for 2 h at RT. Blots were then developed by incubation with ECL chemiluminescence for 1 min (Millipore), and images were captured by Tanon 5200 imaging system. All the antibodies used in this study are listed in Data S1 (Supporting Information).

### Immunofluorescence Staining

For immunofluorescence staining, the cells were seeded onto glass slides in PBS. The slides were placed in a 37 °C incubator for —2 h to evaporate the liquid and then fixed with 4% paraformaldehyde in PBS for 10 min. The paraformaldehyde was removed, and the fixed cells were permeabilized with 0.1% Triton‐X in PBS. The slides were blocked with 1% IgG‐free bovine serum albumin (Sigma‐Aldrich, Cat# A2058) in PBS for 30 min at RT. Cells were incubated with primary antibodies PBS containing 1% bovine serum albumin for 1 h at 37 °C. Slides were then washed three times and incubated with secondary antibodies (Alexa Fluor 488‐conjugated goat anti‐mouse and/or Alex Fluor 594‐conjugated goat anti‐rabbit, diluted 1:250 in PBS) for 1 h at 37 °C. The slides were washed thrice with PBS. An anti‐fade solution containing DAPI was applied to the slide, and then a No. 1.5 coverslip was placed on top. Images were acquired using a LEICA Stellaris STED instrument. Image analysis was performed using LasX software.

For in situ multicolor immunofluorescence staining, a multicolor fluorescence kit (Melabio Biological Technology, Cat# ML0088‐01/ML0088‐05) based on tyramide signal amplification technology was employed to enhance signal detection and improve sensitivity. The tissue chips were incubated for 1 h at 63 °C and dewaxed using a fully automatic dyeing machine. The slides were placed in a repair solution for antigen retrieval, followed by natural cooling. Commercial H_2_O_2_ was used to remove endogenous peroxidase, and blocking buffer was added dropwise. The samples were incubated with primary and secondary antibodies at RT. Samples were stained with opal dye, followed by antibody removal. Other markers were stained before adding an anti‐fade solution containing DAPI, and the coverslips were mounted. Finally, as described previously, the cells were observed and photographed for immunofluorescence staining.

### IHC

IHC was performed on formalin‐fixed, paraffin‐embedded sections of lymph nodes and xenograft tumor specimens using the Benchmark immunohistochemistry staining system (Bond, Leica). The stained sections were imaged using a ZEISS microscope, and the scoring for each antibody was visually estimated at 10% increments. Lymph node specimens in which >30% of tumor cells exhibited SLC3A2 expression were defined as positive (+). Different scores of the positive cases, from + to +++, were recorded according to the depth of the positive staining. The positive percentage score was multiplied by the staining intensity score to generate an immunoreactivity score, with the final score ranging from 0 (minimum score) to 12 (maximum score) for xenograft tumor specimens. The immunoreactivity of each sample was evaluated by two experienced pathologists.

### Quantitative Reverse Transcription PCR (RT‐qPCR)

Total RNA was extracted from the indicated cells and reverse‐transcribed into cDNA using Hiscript III RT SuperMix for qPCR (Vazyme, Cat# R323‐01). qPCR was performed on a CFX96 Real‐Time PCR Detection System (Bio‐Rad) using the Taq Pro Universal SYBR qPCR Master Mix (Vazyme, Cat# Q712‐02). Data were normalized to GAPDH expression. The primer sequences are listed in Table S4 (Supporting Information).

### CRISPR/Cas9 Gene Editing

CRISPR/Cas9 engineering was performed on cells with stable Cas9 expression using Broad Institute Brunello library sgRNA sequences (Table S4, Supporting Information). The sgRNA oligos were cloned into pLentiGuide‐Puro (Addgene, Cat# 52963) or pLentiSpBsmBI sgRNA Hygro (Addgene, Cat# 62205). Lentiviruses were produced in 293T cells by co‐transfection of the target plasmid with psPAX2 and VSVG packaging. At 24 h after transfection, the cell culture medium was changed to RPMI‐1640 + 10% FBS. Two rounds of lentiviral transduction were performed at 48 and 72 h after plasmid transfection. Cells were selected by puromycin (3 µg mL^−1^) or hygromycin (500 µg mL^−1^) added at 48 h after transduction. The depletion of target gene‐encoded proteins was confirmed by immunoblotting.

### cDNA Overexpression

Entry vectors bearing SLC3A2 cDNAs were purchased from Nanjing GeneBay Biological Technology Company. Using homologous recombination, SLC3A2 was cloned into the PCDH‐Hygro vector (Nanjing GeneBay Biological Technology Company). The control vector, PCDH‐Hygro‐GFP, was obtained from Nanjing GeneBay Biological Technology Company. PTCL cell lines were transduced with lentiviruses encoding either PCDH‐Hygro‐GFP or ‐SLC3A2. The cells were selected using hygromycin for 1–2 weeks. Heterogeneous protein expression was confirmed by immunoblotting with an anti‐FLAG‐tagged antibody (ABclonal, Cat# AE092).

### Multicolor Flow Cytometry

To assess the survival and immune receptor expression of T cells isolated from human peripheral blood, T cells cocultured with tumor cells were subjected to flow cytometry. The cells were then incubated with the indicated antibodies in the dark at RT for 30 min. Flow cytometry data were acquired using a Beckman Coulter instrument, and the analysis was performed using FlowJo V10.

### Coculture Experiments

Coculture experiments were performed by seeding 1 × 10^6^ T cells into the upper chamber and 2 × 10^5^ Karpas 299 cells into the bottom chamber of a 24‐well Transwell apparatus with 0.4 mm micropores (Millipore). The cocultured cells were subjected to subsequent analyses after 48 h of incubation.

### ATP Measurements

ATP production was measured using an ATP Determination Kit (Beyotime, Cat# S0026), according to the manufacturer's protocol. Briefly, cells were homogenized in lysis buffer supplemented with a protease and phosphatase inhibitor cocktail. Data collected from multiple replicate wells for each experiment were analyzed using a multimode microplate reader (Agilent, Biotek Synergy H1) and normalized to the number of cells.

### OCR Measurements

The mitochondrial respiratory capacity was determined using the XF Cell Mito Stress Test Kit (Agilent Technologies, Cat #103015–100). PTCL cells were seeded in an XF96 cell culture microplate at a density of 3 × 10^4^ cells per well with replicates of 8–12 with or without arginine. XF96 FluxPak sensor cartridge was hydrated with Seahorse Calibrant overnight in a non‐CO_2_ incubator at 37 °C. The cells were incubated with Seahorse medium for 1 h on the following day. The OCR was measured by a XFe96 extracellular flux analyzer with the sequential injection of 1 µmol L^−1^ oligomycin A, 0.5 µmol L^−1^ FCCP, and 0.5 µmol L^−1^ rotenone/antimycin A. After the experiment, the OCR value in each well was normalized to the protein concentration determined using the Pierce BCA Protein Assay Kit (Thermo Fisher Scientific, Cat# 23225).

### ATAC‐seq and ChIP‐qPCR

For the ATAC assays, 1 × 10^5^ cells were harvested following the manufacturer's protocol using the TruePrep DNA Library Prep Kit V2 for Illumina (Vazyme, Cat# TD501). Samples were sequenced on an Illumina NovaSeq 5000 platform using standard procedures. Sequenced reads were aligned to the Hg38 human reference genome using Bowtie2 (v 2.4.2), and the generated SAM files were converted into BAM files using the Samtools (v 1.11) view function. Samblaster (v 0.1.26) was used to further sort, index, and remove potential PCR duplicates (rmdup) from the BAM files, which were converted to BigWig files using Deeptools v3.5.3. BigWig files were visualized using the Integrative Genomics Viewer (IGV; Broad Institute).

For ChIP‐qPCR, the Sonication ChIP Kit (ABclonal Cat# RK20258) was used according to the manufacturer's instructions. Briefly, the indicated cells were cross‐linked with 1% formaldehyde, and the reaction was quenched with glycine. The fixed cells were harvested, lysed, and sonicated on ice. The indicated antibodies were used for immunoprecipitation. Rabbit IgG was used as a negative control. Ten percent of the chromatin sample before immunoprecipitation was used as an input control. The precipitated DNA was then subjected to qPCR. The primer sequences used for ChIP‐qPCR are listed in Table S4 (Supporting Information).

### co‐IP

To detect SSRP1 and JUNB interactions, a Sonication ChIP Kit (Abclonal, Cat# RK20258) was used for a co‐IP assay according to the manufacturer's instructions. Briefly, cells were lysed without cross‐linking using lysis buffer supplemented with a protease and phosphatase inhibitor cocktail. Anti‐SSRP1 or anti‐JUNB antibodies were mixed with Protein G Sepharose for each sample and incubated at 4 °C for 1 h with constant rotation. Rabbit or mouse IgG served as a negative control. The antibody–Sepharose conjugates were washed once with lysis buffer, added to the soluble fraction of centrifuged cell lysates from each sample, and incubated at 4 °C for 2 h with constant rotation. The immunoprecipitates were washed thrice with lysis buffer and boiled with SDS loading buffer, followed by western blot analysis.

### siRNA‐Mediated Knockdown

H9 cells were seeded in a 6‐well plate and transfected with siRNAs using Lipo3000 (Thermo Fisher Scientific, Cat# L3000015) in OptiMEM for siRNA‐mediated knockdown. The cells were subjected to subsequent analyses after 48 h of incubation. The siRNA sequences are listed in Table S4 (Supporting Information).

### MALDI‐MSI

NSG mice bearing xenografts were treated with quinacrine (20 mg kg^−1^ i.p. QOD) for 6 days. Mice were euthanized 4 h after the last dose. Intact tumor samples were collected and processed for MALDI‐MSI analysis. The procedures was as follows:

iv) Sample Preparation (frozen section)

The frozen tissue samples were fixed in three drops of distilled water during the cutting stage. The tissues were sectioned at 12 µm thickness using a Leica CM1950 cryostat (Leica Microsystems GmbH, Wetzlar, Germany) at −20 °C. Afterward, the tissue sections were placed in groups on electrically conductive slides coated with indium tin oxide (ITO), and the slides with tissue sections were dried in a vacuum desiccator for 30 min.

v) Matrix Coating

Desiccated tissue sections mounted on ITO glass slides were sprayed with 15 mg mL^−1^ DHB (2, 5‐dihydroxybenzoic acid) dissolved in 9:1 acetonitrile:water using an HTX TM sprayer (Bruker Daltonics, Germany). The sprayer temperature was set to 60 °C, with a flow rate of 0.12 mL min^−1^ and a pressure of 6 psi. Twenty‐two passes of the matrix were applied to the slides with a drying time of 5 s between each pass.

vi) Mass Spectrometry Imaging

MALDI‐TOF MSI experiments were performed on a prototype Bruker timsTOF flex MS system (Bruker Daltonics, Bremen, Germany) equipped with a 10 kHz smart beam 3D laser. The laser power was fixed at 80% throughout the experiment. Mass spectra were acquired in positive mode. Mass spectral data were obtained over the mass range of m/z 50–1300 Da. The imaging spatial resolution was set to 100 × 10^−6^ m for the tissue, and each spectrum consisted of 400 laser shots. MALDI mass spectra were normalized using the root mean square, and the signal intensity in each image is shown as the normalized intensity. MS/MS fragmentations performed on the timsTOF flex MS system in the MS/MS mode were employed for further detailed structural confirmation of the identified metabolites.

### In Vivo Experiments

Four‐ to six‐week‐old NCG mice were injected subcutaneously into the armpit of the forelimb of each flank with 10 million SU‐DHL‐1 cells in PBS with an injection volume of 200 µL. Once palpable tumors were established, the tumor volume and body weight were measured every 2 or 3 days. Tumor volume was calculated using the following formula: volume = length × (width)^2^/2. Once tumors were established with a volume of 100 mm^3^, the mice were randomized into groups of to 4–9 individuals.

Trial 1: As illustrated in Figure 1N, mice were randomized into vehicle and treatment groups. A single injection of quinacrine (dosing concentration, 20 mg kg^−1^) or vehicle control was administered intraperitoneally. Half of the animals were euthanized (*n* = 4 quinacrine, *n* = 4 vehicle control) after day 6 of administration for MALDI‐MSI analysis and the other half on day 9.

Trial 2: As illustrated in Figure 4S, the mice were subcutaneously injected with SU‐DHL‐1 cells transduced with sg*Control* or sg*SLC3A2* to generate SLC3A2‐knock out PTCL xenografts.

Trial 3: As illustrated in Figure 5L, mice used in the arginine‐free diet experiments were divided into two groups. One group was fed a control diet and the second group was fed an arginine‐free diet. The diet was initiated 1 week prior to tumor inoculation.

Trial 4: As illustrated in Figure 7D, quinacrine (20 mg kg^−1^, i.p.) was administered to mice in the quinacrine monotherapy and combination therapy groups every 2 days, whereas chidamide (15 mg kg^−1^, oral gavage) was administered to mice in the chidamide monotherapy and combination therapy groups every day during the experiment.

Tumors were collected, flash‐frozen in liquid nitrogen, or fixed in 10% formalin for downstream analyses. All animals were euthanized via CO_2_ inhalation at a flow rate of 3.5 L min^−1^.

### Statistical Analysis

All experimental data were pre‐processed before statistical analysis. Gene expression matrix was normalized and scaled using NormalizeData and ScaleData. Data in the graphs are shown as mean ± standard deviation (SD) unless otherwise noted. The sample sizes (*n*) for each statistical analysis were included in the figure legends. *p* values between two groups were determined using Student's *t*‐test, Pearson's chi‐square test, Fisher's exact test, or Wilcoxon rank‐sum test, while for experiments with more than two groups, *p* values were determined by one‐ or two‐way ANOVA. For survival analysis, the log‐rank test was used to calculate *p* values between groups, and the Kaplan–Meier method was used to plot survival curves. Pearson's correlation coefficient was calculated to assess the association between two continuous variables at the sample level. Details of the statistical analyses are provided in the figure legends. A *p* value <0.05 was considered statistically significant. All statistical tests were two‐sided. Statistical analyses were performed using Prism (v9.0, GraphPad), SPSS (v26.0, IBM), and R (v4.0.0) software.

## Conflict of Interest

The authors declare no conflict of interest.

## Supporting information



Supporting Information

## Data Availability

Any information required to reanalyze the data reported in this paper is available from the lead contact upon request (jinhui@jsph.org.cn).
