# Supplementary material for: SSRP1/SLC3A2 Axis in Arginine Transport: A New Target for Overcoming Immune Evasion and Tumor Progression in Peripheral T‐Cell Lymphoma
Source: Adv Sci (Weinh). 2025 May 8;12(21):2415698. doi: 10.1002/advs.202415698 (PMC12140342; doi:10.1002/advs.202415698)
Supplement: Supplementary file 1 — Supporting Information [file ADVS-12-2415698-s001.docx]

Supporting Information

**The SSRP1/SLC3A2 Axis in Arginine Transport: A New Target for Overcoming Immune Evasion and Tumor Progression in Peripheral T-cell Lymphoma**

*Yimin Ren, Lei Fan^*^, Ling Wang, Yanping Liu, Jie Zhang, Boya Wang, Ruize Chen, Xiao Chen, Lingyu Zhuang, Yaping Zhang, Handong Sun^*^,Jianyong Li^*^, Wenyu Shi^*^, Hui Jin^*^*

This file includes:

Figures. S1 to S7

Tables. S1 to S4

Data S1 (Key resource table)

Figure S1


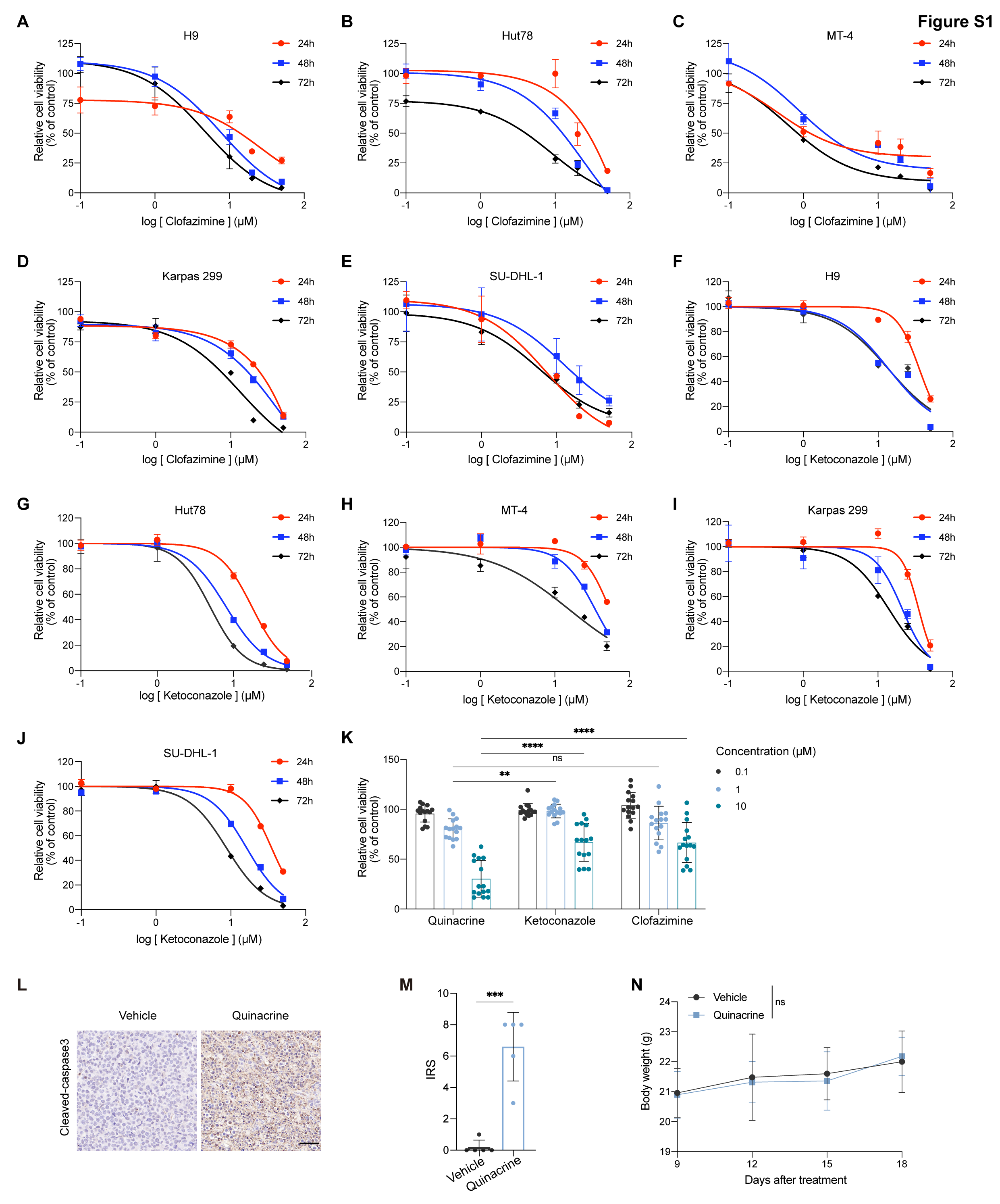


**Figure S1. *In vitro* HTS identifies quinacrine as a potential drug in cell models of PTCL**

**(A–E)** Relative cell viability of H9 (A), Hut78 (B), MT-4 (C), Karpas 299 (D) and SU-DHL-1 (E) treated with different concentrations of clofazimine for 24, 48, and 72 h (n = 3).

**(F–J)** Relative cell viability of H9 (F), Hut78 (G), MT-4 (H), Karpas 299 (I) and SU-DHL-1 (J) treated with different concentrations of ketoconazole for 24, 48, and 72 h (n = 3).

**(K)** Scatter plot of the relative cell viability of the three hits at different concentrations (0.1, 1, and 10μM) across PTCL cell lines (n = 3).

**(L, M)** Representative IHC staining (L) showing cleaved-caspase3 expression in tumors treated with vehicle or quinacrine, and the corresponding IRS scores (M) (n = 5). Scale bar, 50 μM.

**(N)** Body weight measurements of mice subcutaneously injected with SU-DHL-1 cells treated with either quinacrine or vehicle (n=5).

For all panels, the data are presented as means ± SD. *, *P* < 0.05; **, *P* < 0.01; ***, *P* < 0.001; ****, *P* < 0.0001; ns, non-significant. For K, *P* values were generated using two-way ANOVA with multiple comparisons. For M and N, *P* values were generated using Student’s two-tailed unpaired t test.

Figure S2


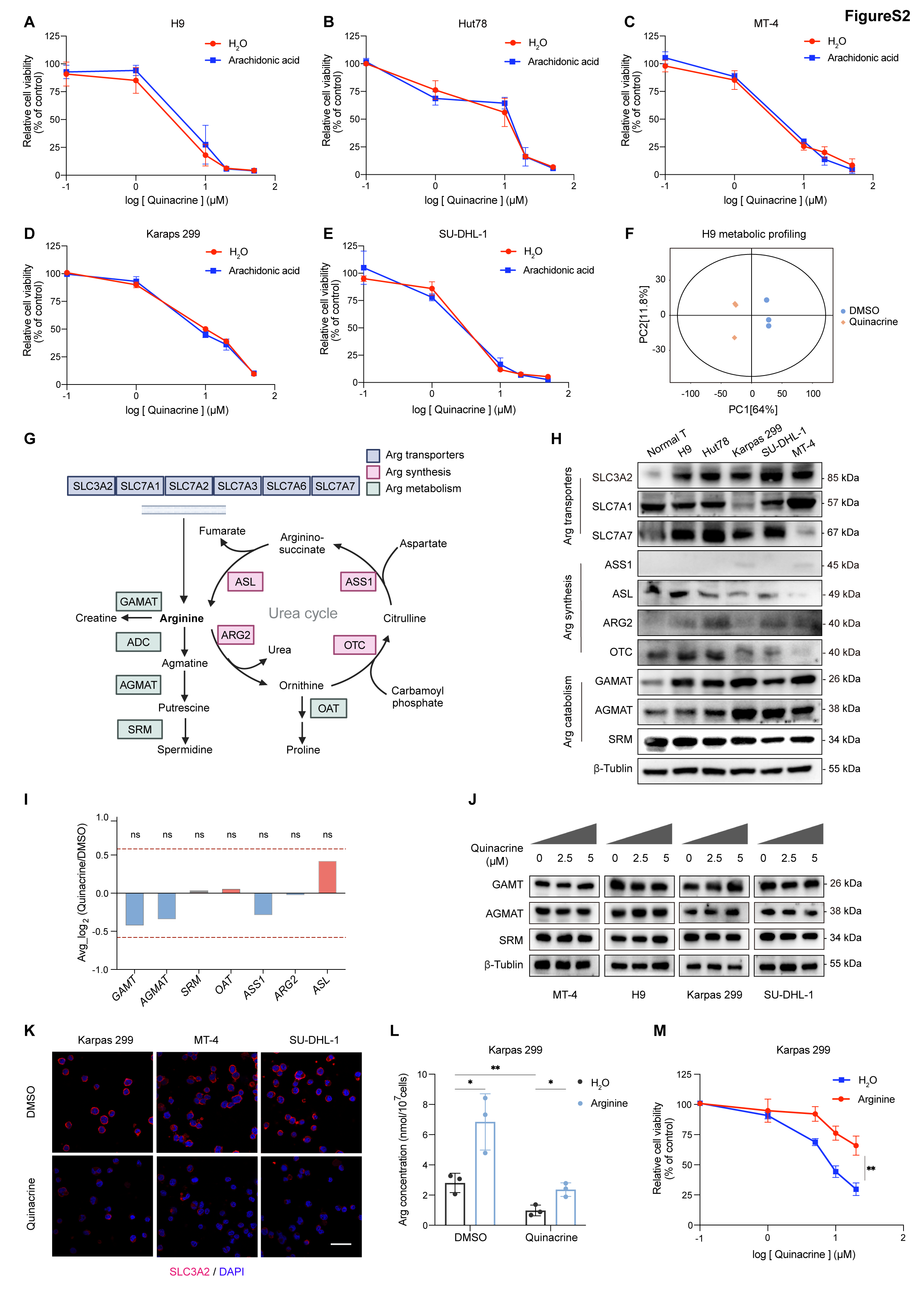


**Figure S2. Metabolomic and transcriptomic analyses reveal that quinacrine downregulates the arginine transporter SLC3A2 and causes arginine deficiency**

**(A–E)** Relative cell viability of H9 (A), Hut78 (B), MT-4 (C), Karpas 299 (D) and SU-DHL-1 (E) treated with different concentrations of quinacrine and cultured in H_2_O or arachidonic acid (40 μM) for 48 h (n = 3).

**(F)** PCA of intracellular metabolites, as determined by LC-MS/MS in quinacrine-treated (light red) or DMSO- (light blue) H9 cells (n = 3). DMSO, dimethyl sulfoxide.

**(G)** Schematic representation of the arginine metabolism pathway.

**(H)** Immunoblots of the indicated proteins in PTCL cell lines and normal T cells isolated from healthy volunteers (n = 3). β-Tubulin serves as a loading control.

**(I)** Bar plot showing average fold change of indicated genes in H9 cells treated with quinacrine versus DMSO for 48 h.

**(J)** Immunoblots of the indicated proteins in PTCL cell lines treated with different concentrations of quinacrine for 48h (n = 3). β-Tubulin serves as a loading control.

**(K)** Representative SLC3A2 immunofluorescence staining of MT-4, Karpas 299 and SU-DHL-1 cells treated with quinacrine or DMSO (2 μM) for 48h (n = 3). Scale bar, 25 μM.

**(L)** Arginine levels in Karpas 299 cells treated with quinacrine (2 μM) or DMSO and cultured in H_2_O or arginine (4 mM) for 48h (n = 3).

**(M)** Relative cell viability of H9 cells treated with different concentrations of quinacrine and cultured in H_2_O or arginine (4 mM) for 48h (n = 3).

For all panels, the data are presented as mean ± SD. *, *P* < 0.05; **, *P* < 0.01; ***, *P* < 0.001; ****, *P* < 0.0001; ns, non-significant. For I, *P* values were generated using Wilcoxon rank-sum test with default parameters. For L, *P* values were generated using two-way ANOVA with multiple comparisons. For M, *P* values were generated using Student’s two-tailed unpaired t test.

Figure S3


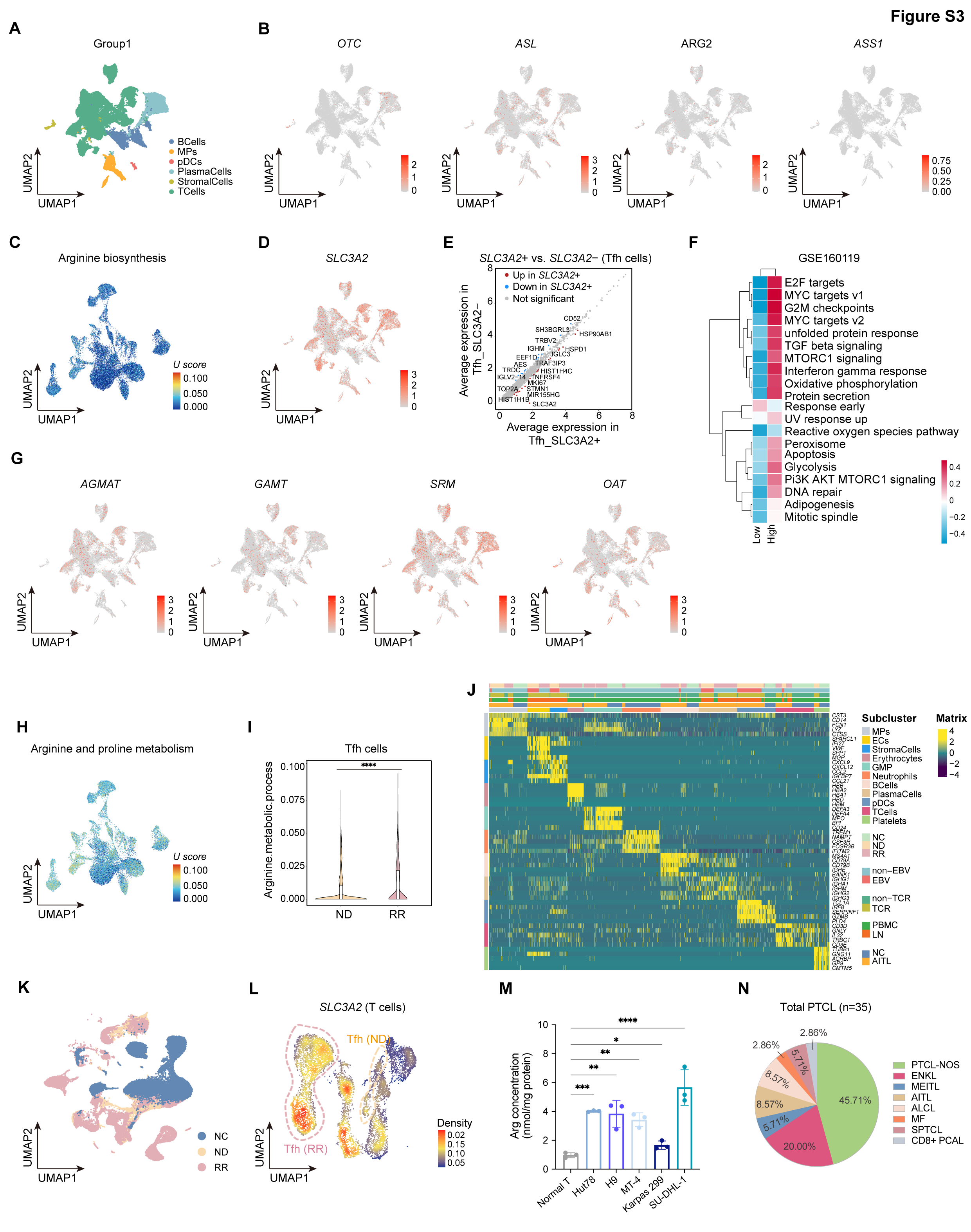


**Figure S3. Characterization of arginine metabolism in patients with PTCL**

1. UMAP plot of six cell types in nine specimens from Group1.
2. UMAP plots of the expression of four key enzymes *OTC*, *ASL*, *ARG2* and *ASS1* (left to right) in urea cycle in all cell types.
3. UMAP plot of arginine biosynthesis scores based on KEGG pathway enrichment (has00220) of all cell types.
4. UMAP plot of the expression of *SLC3A2* in all cell types.

Volcano plot showing DEGs in *SLC3A2*+ Tfh cells versus *SLC3A2*− Tfh cells. Top20 DEGs are labeled.

1. Heatmap of significantly enriched pathways in *SLC3A2*_high versus *SLC3A2*_low patients with PTCL from GSE160119.
2. UMAP plots of the expression of four key enzymes *AGMAT, GAMT, SRM* and *OAT* (left to right) in arginine catabolism pathway in all cell types.
3. UMAP plot of arginine and proline metabolism scores based on KEGG pathway enrichment (hsa00330) of all cell types.
4. Violin plot showing arginine metabolic process scores based on GO: 0006525 of Tfh tumor cells in ND and RR group from Group2.
5. Heatmap showing the expression of five representative marker genes in each cell type from Group2.
6. UMAP of three groups (NC, ND, and RR) identified in Group2.
7. The density plot of *SLC3A2* expression shown in the UMAP visualization of T cells from Group2. Tfh tumor cells are indicated by dashed circles.
8. Arginine levels detected in normal T cells and indicated cell lines (n = 3).
9. Pie chart showing the proportion of eight different PTCL subtypes.

For all panels, data are presented as mean ± SD. *, *P* < 0.05; **, *P* < 0.01; ***, *P* < 0.001; ****, *P* < 0.0001; ns, non-significant. For I, *P* value was generated using Wilcoxon rank-sum test with default parameters. For M, *P* values were generated using one-way ANOVA with multiple comparisons.

Figure S4


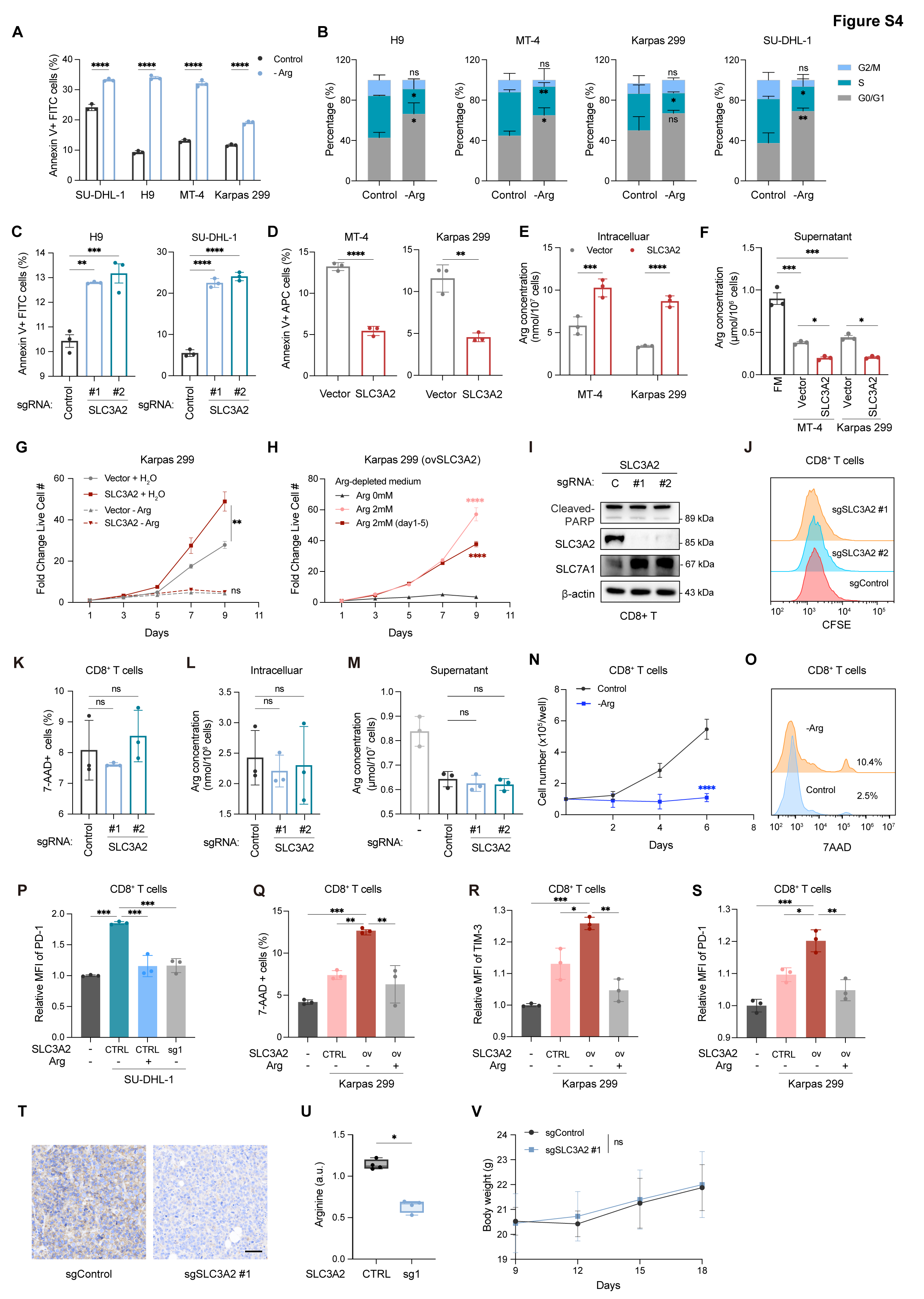


**Figure S4. PTCL relies on SLC3A2-mediated arginine uptake to ensure proliferation**

1. Cell apoptosis assays of the four PTCL cell lines under control and arginine-depleted (-Arg) conditions (n = 3).
2. Cell cycle analysis of the four PTCL cell lines under control and arginine-depleted conditions (n = 3).
3. Cell apoptosis assays of H9 (left) and SU-DHL-1 (right) cells at day 7 after sgRNAs expression (n = 3).
4. Cell apoptosis assays of MT-4 (left) and Karpas 299 (right) cells expressing a vector (control) or SLC3A2 cDNA (n = 3).

**(E, F)** Arginine levels in cell lysates (E) and supernatants (F) of the indicated cells. FM, fresh medium (n = 3).

**(G, H)** Growth curves of Karpas 299 cells after the indicated treatments (n = 3).

1. Immunoblots of indicated proteins in Cas9+ CD8^+^ T cells expressing non-targeting control or independent sgRNAs against *SLC3A2*. Whole cell lysate was obtained at day 7 after sgRNA expression (n = 3). β-actin serves as a loading control.
2. Flow cytometric analysis of CFSE in Cas9+ CD8^+^ T cells expressing non-targeting control or independent sgRNAs against *SLC3A2* (n = 3).
3. Flow cytometric analysis of 7-AAD in Cas9+ CD8^+^ T cells expressing non-targeting control or independent sgRNAs against *SLC3A2* (n = 3).

**(L, M)** Arginine levels in cell lysates (L) and supernatants (M) of Cas9+ CD8^+^ T cells expressing non-targeting control or independent sgRNAs against *SLC3A2* (n = 3).

1. Growth curves of CD8^+^ T cells under control and arginine-depleted conditions (n = 3).
2. Flow cytometric analysis of 7-AAD-positive CD8^+^ T cells under control and arginine-depleted conditions (n = 3).
3. Flow cytometric analysis of relative MFI of PD-1 on CD8^+^ T cells co-cultured with SLC3A2-WT or SLC3A2 knockout SU-DHL-1 cells and/or arginine addition (n = 3).
4. Flow cytometric analysis of 7-AAD-positive CD8^+^ T cells co-cultured with SLC3A2-WT or SLC3A2 overexpression Karpas299 cells and/or arginine addition (n = 3).
5. Flow cytometric analysis of relative MFI of TIM-3 on CD8^+^ T cells co-cultured with SLC3A2-WT or SLC3A2 overexpression Karpas299 cells and/or arginine addition (n = 3).
6. Flow cytometric analysis of relative MFI of PD-1 on CD8^+^ T cells co-cultured with SLC3A2-WT or SLC3A2 overexpression Karpas299 cells and/or arginine addition (n = 3).
7. Representative IHC staining showing SLC3A2 expression in the subcutaneous tumors of the SU-DHL-1 xenograft mouse model expressing control or *SLC3A2* sgRNAs (n = 4). Scale bar, 50 μM.
8. Arginine levels in the subcutaneous tumors of the SU-DHL-1 xenograft mouse model expressing control or *SLC3A2* sgRNAs detected by LC-MS/MS. Control group levels were set to 1 (n = 4).
9. Body weight measurements of the SU-DHL-1 xenograft mice expressing control or *SLC3A2* sgRNAs (n = 4).

For all panels, the data presented are means ± SD. *, *P* < 0.05; **, *P* < 0.01; ***, *P* < 0.001; ****, *P* < 0.0001; ns, non-significant. For A, B, D, E, I, U and V, *P* values were generated using Student’s two-tailed unpaired t test. For C, F-H and M-S, *P* values were generated using one-way ANOVA with multiple comparisons.

Figure S5


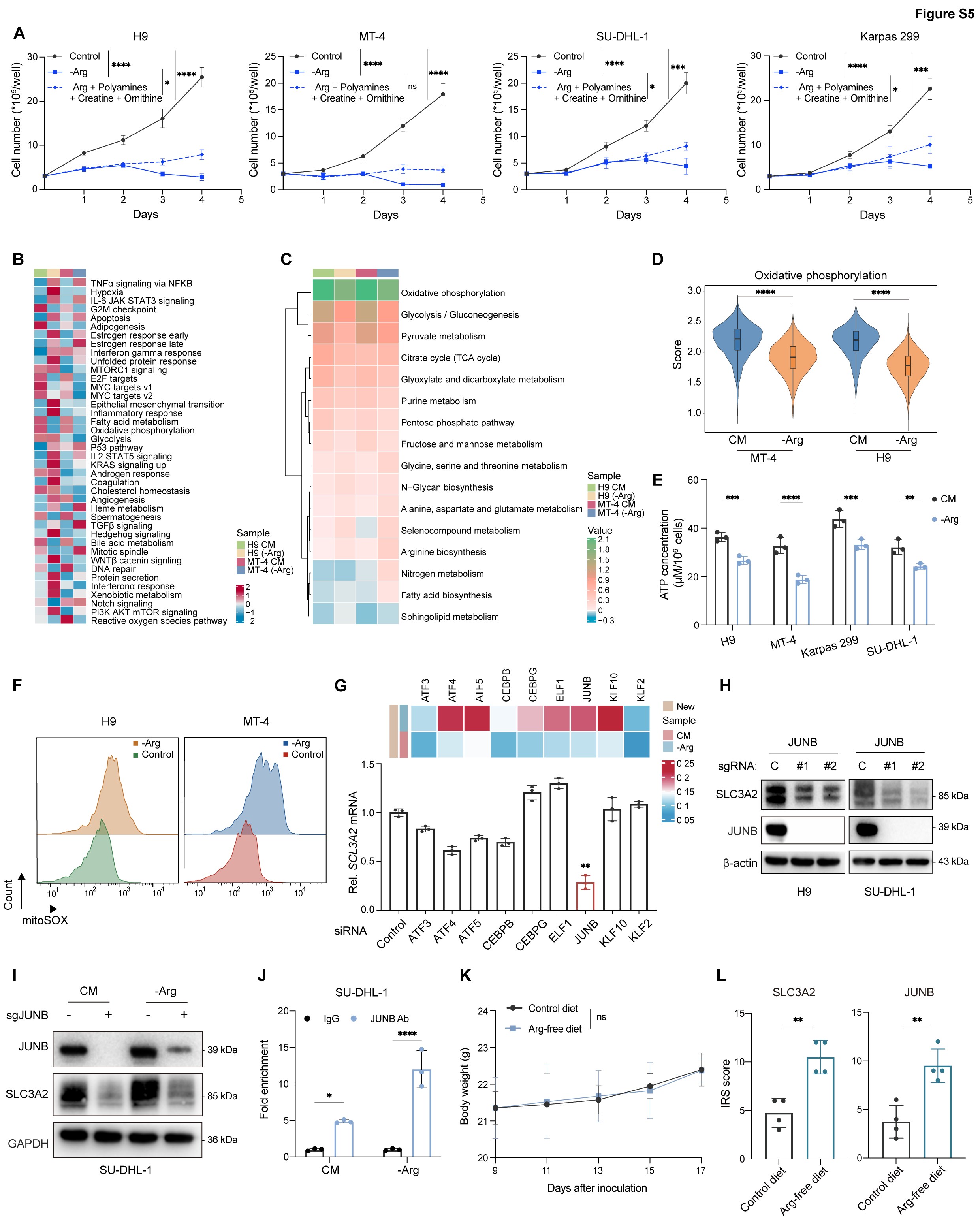


**Figure S5. Single-cell dynamic RNA sequencing reveals that arginine deficiency reduces OXPHOS and induces nascent RNA of SLC3A2 via JUNB**

1. Growth curves of PTCL cell lines treated with three polyamines (50 μM putrescine, 5 μM spermidine, 5 μM spermine), 1 mM ornithine, and 1 mM creatine under control and arginine-depleted (-Arg) conditions (n = 3).
2. Heatmap of hallmark analysis in the arginine deprivation group compared to complete medium (CM) group according to total RNAs.
3. Heatmap of scMetabolism analysis in arginine deprivation group compared to complete medium group according to total RNAs.
4. Violin plot of scMetabolism scores of OXPHOS pathway in H9 and MT-4 cells incubated in complete or arginine depleted medium.
5. Intracellular ATP levels in control and arginine-depleted PTCL cells at 48 h (n = 3).
6. Flow cytometric analysis of mitoSOX in H9 and SU-DHL-1 cells incubated in complete or arginine depleted medium (n = 3).
7. Heatmap showing regulation activity of 9 upregulated TFs that potentially bind to the SLC3A2 promoter in new RNAs of arginine depleted group versus control group (top). qPCR (bottom) showing *SLC3A2* expression levels in H9 cells transduced with indicated siRNA (n = 3).
8. Immunoblots of SLC3A2 expression in Cas9+ PTCL cell lines expressing non-targeting control or independent sgRNAs against *JUNB*. Whole-cell lysates were obtained at day 7 after sgRNA expression (n = 3). β-actin serves as a loading control.
9. Immunoblots of whole cell lysates of Cas9+ SU-DHL-1 cells expressing non-targeting control or independent sgRNAs against *JUNB* in complete or arginine depleted medium (n = 3). GAPDH serves as a loading control.
10. Nuclear extracts of SU-DHL-1 cells incubated in complete or arginine depleted medium were immunoprecipitated and analyzed by ChIP (n = 3). IgG serves as a control.
11. Body weight measurements of the SU-DHL-1 xenograft mice fed with control and arginine-free diet (n = 4).
12. Quantification of JUNB and SLC3A2 expression from tumors of control and arginine-free diet-fed mice (n = 4).

For all panels, the data are presented as means ± SD. *, *P* < 0.05; **, *P* < 0.01; ***, *P* < 0.001; ****, *P* < 0.0001; ns, non-significant. For A, *P* values were generated by were generated by one-way ANOVA with multiple comparisons. For D, *P* values were generated using Wilcoxon rank-sum test. For E, G, K, L, *P* values were generated using Student’s two-tailed unpaired t test. For J, *P* values were generated using two-way ANOVA with multiple comparisons.

Figure S6


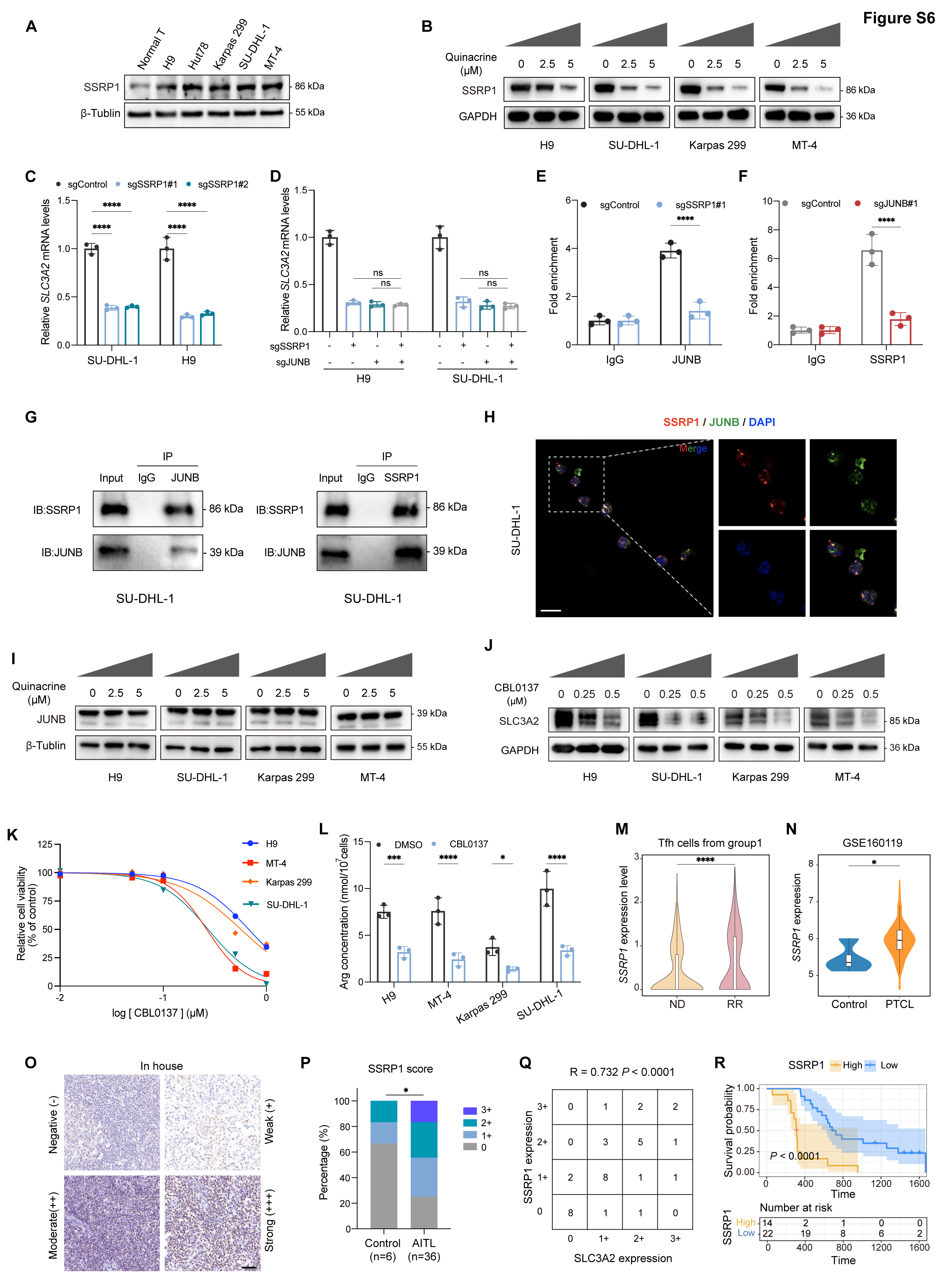


**Figure S6. Quinacrine transcriptionally regulates SLC3A2 by targeting SSRP1 in a JUNB-dependent manner**

1. Immunoblots of SSRP1 in PTCL cell lines and normal T cells isolated from healthy volunteers (n = 3). β-Tubulin serves as a loading control.
2. Immunoblots of SSRP1 in PTCL cell lines treated with different concentrations of quinacrine for 48h (n = 3). GAPDH serves as a loading control.
3. RT-qPCR analysis of *SLC3A2* mRNA levels in H9 and SU-DHL-1 cells expressing the indicated sgRNAs for 5 days (n = 3).
4. RT-qPCR analysis of mRNA expression levels of *SLC3A2* in H9 and SU-DHL-1 cells expressing control sgRNA or *SSRP1* sgRNA, together with or without *JUNB* sgRNA (n = 3).
5. A ChIP assay was performed in SU-DHL-1 cells expressing control or *SSRP1* sgRNAs using anti-JUNB or anti-IgG antibodies, followed by RT-qPCR. The fold cahnge in the expression of ChIP-enriched mRNAs relative to the input was calculated (n = 3). IgG serves as a control.
6. A ChIP assay was performed in SU-DHL-1 cells expressing control or *JUNB* sgRNAs using anti-SSRP1 or anti-IgG antibodies, followed by RT-qPCR. The fold change in the expression of ChIP-enriched mRNAs relative to the input was calculated (n = 3). IgG serves as a control.
7. Immunoblots of SSRP1 and JUNB from anti-JUNB IPs (left) and anti-SSRP1 IPs (right) obtained from SU-DHL-1 cells (n = 3).
8. Representative immunofluorescence staining of JUNB and SSRP1 in SU-DHL-1 cells (n = 3). Scale bar, 25 μM.
9. Immunoblots of JUNB in PTCL cell lines treated with different concentrations of quinacrine for 48h (n = 3). β-Tubulin serves as a loading control.
10. Immunoblots of SLC3A2 in PTCL cell lines treated with different concentrations of CBL0137 for 48 h (n = 3). β-Tubulin serves as a loading control.
11. Relative cell viability of H9, MT-4, Karpas 299, and SU-DHL-1 treated with different concentrations of CBL0137 for 48 h (n = 3).
12. Arginine levels in PTCL cell lines treated with CBL0137 (0.25 μM) or DMSO for 48 h (n = 3).
13. Violin plot showing the *SSRP1* expression in Tfh tumor cells in the ND and RR group from Group1.
14. Violin plot showing the *SSRP1* expression in NC and PTCL tissues from GSE160119.
15. Patterns of different levels of SSRP1 expression in normal lymph nodes and AITL tissues detected by IHC analysis. Scale bar, 50 μM.
16. The distribution of different expression levels of SSRP1 in tissues from 36 AITL patients and 6 NCs.
17. The correlation of SSRP1 and SLC3A2 protein expression detected by IHC in 36 AITL patients.
18. Kaplan–Meier curves of OS in AITL patients with different SSRP1 expression levels in our cohort.

For all panels, data are presented as means ± SD. *, *P* < 0.05; **, *P* < 0.01; ***, *P* < 0.001; ****, *P* < 0.0001; ns, non-significant. For C, D, *P* values were generated using one-way ANOVA with multiple comparisons. For E, F, *P* values were generated using two-way ANOVA with multiple comparisons. For L, *P* values were generated using Student’s two-tailed unpaired t test. For M and N, *P* values were generated using Wilcoxon rank-sum test. For P, *P* value was generated using Fisher's exact test. For Q, *P* value was generated using Pearson’s test. For R, *P* value was generated by the log-rank test.

Figure S7


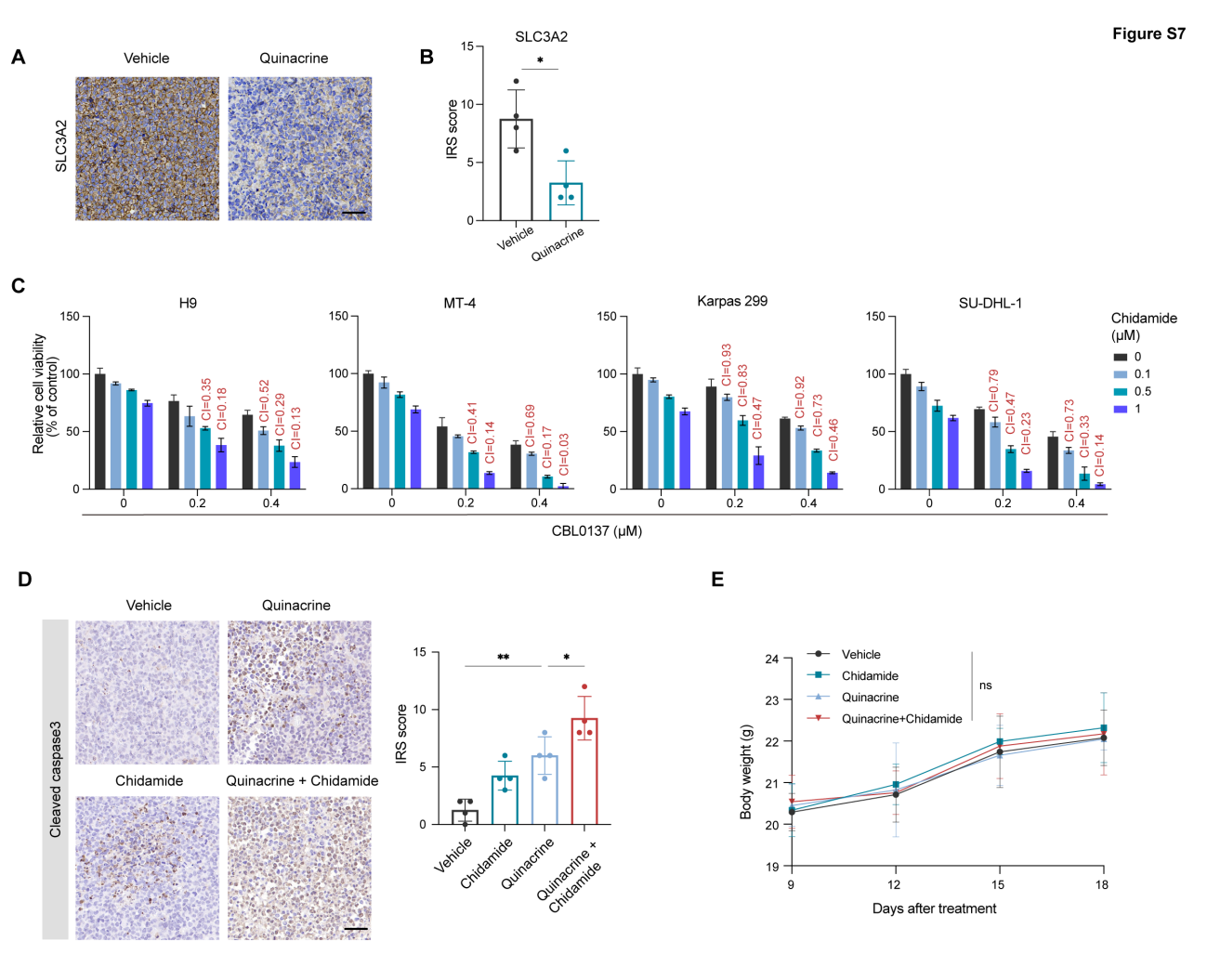


**Figure S7. Combination epigenetic therapy exerts a synergistic antitumor effect on PTCL**

**(A, B)** Representative images (A) of IHC showing SLC3A2 expression in tumor samples of the two groups (vehicle and quinacrine) and the corresponding quantification (B) (n = 4). Scale bar, 50 μM.

1. CCK8 analysis of the relative proliferation activity of the PTCL cell lines treated with CBL0137 in combination with chidamide at different concentrations for 48 h (n = 3). The CI is red-coded.
2. Representative images (left) of IHC showing Ki-67 expression in tumor samples of the four groups (vehicle, chidamide, quinacrine, and quinacrine plus chidamide groups) and corresponding quantification (right) (n = 4). Scale bar, 50 μM.
3. Body weight measurements of mice subcutaneously injected with SU-DHL-1 cells treated with vehicle, chidamide, quinacrine, and quinacrine in combination with chidamide (n = 4).

For all panels, data are presented as mean ± SD. *, *P* < 0.05; **, *P* < 0.01; ***, *P* < 0.001; ****, *P* < 0.0001; ns, non-significant. For B, *P* value was generated by Student’s two-tailed unpaired t test. For D and E, *P* values were generated by one-way ANOVA with multiple comparisons.

**Supplemental Tables**

**Table S1. Results of Metabolic Inhibitor Library Screen.**

| Compound | Cell inhibition rate (average) | | | | | | |  |
| --- | --- | --- | --- | --- | --- | --- | --- | --- |
|  | H9 | | Hut78 | | Karpas 299 | | MT-4 | |
| Amino acid_Carbidopa | 42.33% | -8.19% | | 41.40% | | 78.44% | | |
| Amino acid_Benserazide HCl | 47.93% | -6.37% | | 36.18% | | 81.35% | | |
| Amino acid_Eflornithine hydrochloride hydrate | -10.27% | 5.22% | | 8.15% | | -8.42% | | |
| Amino acid_LX 1606 Hippurate | 51.25% | 4.78% | | -12.10% | | 93.23% | | |
| Amino acid_Sapropterin Dihydrochloride | -27.86% | 5.17% | | 2.82% | | 10.74% | | |
| Amino acid_Indoximod (NLG-8189） | 55.04% | -16.16% | | 22.36% | | 50.07% | | |
| Amino acid_Vitamin K1 | 5.00% | 2.20% | | 2.82% | | 8.99% | | |
| Amino acid_Baicalein | -13.71% | 0.00% | | 15.38% | | 81.24% | | |
| Amino acid_Vitamin B12 | 3.70% | 12.00% | | 18.07% | | -31.37% | | |
| Amino acid_Pyridoxine HCl | 4.17% | 13.16% | | 2.92% | | -8.57% | | |
| Amino acid_Pyridoxine | 1.59% | 7.22% | | 7.26% | | -5.10% | | |
| Amino acid_Pyridoxal 5-phosphate monohydrate | 0.81% | -1.08% | | 26.85% | | 74.52% | | |
| Amino acid_Methylcobalamin | 16.82% | 12.05% | | 8.05% | | 5.04% | | |
| Amino acid_Pyridoxal phosphate | 8.97% | 3.02% | | 5.56% | | -51.15% | | |
| Amino acid_VitaMin U | -21.33% | 7.37% | | -5.28% | | -5.04% | | |
| Central carbon_Enasidenib | 15.98% | -9.30% | | 20.12% | | 0.83% | | |
| Central carbon_Ivosidenib | 4.68% | 12.01% | | 8.92% | | -25.33% | | |
| Central carbon_Sodium dichloroacetate | 0.31% | 0.55% | | 5.43% | | -28.94% | | |
| Central carbon_AG-120 | 5.41% | 9.20% | | -0.08% | | 5.82% | | |
| Central carbon_Pioglitazone HCl | -5.63% | -20.00% | | -6.31% | | -2.58% | | |
| Central carbon_DL-Serine | -1.82% | 20.76% | | -5.83% | | -10.00% | | |
| Central carbon_Troglitazone | 7.74% | 6.87% | | 1.01% | | 15.16% | | |
| Central carbon_Harmine | 61.45% | 14.71% | | 25.60% | | 74.79% | | |
| Central carbon_D panthenol | 3.71% | -1.03% | | 11.09% | | -26.29% | | |
| Central carbon_D-Pantothenate Sodium | -24.33% | 5.31% | | -5.34% | | -57.01% | | |
| Central carbon_Fursultiamine | -11.00% | -1.91% | | 19.17% | | 62.18% | | |
| Central carbon_Thiamine HCl | -13.00% | 12.94% | | -4.65% | | 38.48% | | |
| Central carbon_D-Pantethine | -11.67% | 9.57% | | -5.38% | | -9.25% | | |
| Choline_Edrophonium chloride | -2.92% | -19.00% | | 0.54% | | -3.59% | | |
| Choline_Cevimeline | -1.71% | 9.60% | | 5.45% | | 4.76% | | |
| Choline_Irsogladine | 51.05% | -4.49% | | 19.38% | | 54.77% | | |
| Choline_Atropine sulfate | -4.67% | 1.97% | | -3.61% | | 28.37% | | |
| Choline_Meclofenoxate (Centrophenoxine) HCl | 0.31% | 0.67% | | 6.29% | | -70.01% | | |
| Detooxification_Mafenide Acetate | 2.89% | -5.48% | | -5.93% | | 24.27% | | |
| Detooxification_Mafenide hydrochloride | 3.92% | -0.94% | | 1.61% | | -56.81% | | |
| Detooxification_Posaconazole | -8.33% | 3.33% | | 2.16% | | 1.94% | | |
| Detooxification_Fluconazole | 0.67% | 3.67% | | 3.17% | | -40.97% | | |
| Detooxification_Voriconazole | -3.27% | 6.51% | | -5.37% | | 15.99% | | |
| Detooxification_Thiabendazole | 6.34% | 15.72% | | 0.15% | | 5.87% | | |
| Detooxification_Apigenin | -11.90% | 10.33% | | 2.01% | | 25.14% | | |
| Detooxification_Clarithromycin | 5.51% | 43.64% | | -6.85% | | 20.88% | | |
| Detooxification_Sulfaphenazole | -17.70% | 1.07% | | 8.05% | | 4.08% | | |
| Detooxification_Itraconazole | 38.03% | 25.81% | | 24.16% | | 69.18% | | |
| Folate_Methotrexate | 3.17% | -8.85% | | 23.25% | | 0.40% | | |
| Folate_Pralatrexate | -2.59% | -8.15% | | 27.85% | | -2.19% | | |
| Folate_Sulfameter | 83.43% | 12.02% | | 14.42% | | -2.89% | | |
| Folate_Pyrimethamine | -0.24% | -6.98% | | 10.69% | | 16.51% | | |
| Folate_Diaveridine | -3.16% | 4.16% | | -3.70% | | 28.04% | | |
| Folate_Pemetrexed Disodium Hydrate | -2.22% | -7.69% | | -5.93% | | 25.48% | | |
| Folate_Methotrexate disodium | -0.34% | 10.03% | | -1.03% | | -10.51% | | |
| Folate_Pemetrexed | -3.26% | -4.38% | | 11.22% | | 17.00% | | |
| Folate_Folic acid | 33.67% | -8.36% | | 24.36% | | 57.57% | | |
| Hormone_Aspirin | 9.60% | 6.88% | | 5.16% | | -78.98% | | |
| Hormone_Oxaprozin | 2.78% | -1.36% | | -4.70% | | -64.12% | | |
| Hormone_Enoxolone | 15.48% | -4.52% | | -13.36% | | -9.55% | | |
| Hormone_Ammonium Glycyrrhizinate | -8.71% | -1.42% | | -0.03% | | -3.89% | | |
| Hormone_Glycyrrhizin (Glycyrrhizic Acid) | -2.52% | 6.60% | | -0.94% | | 9.42% | | |
| Hormone_Osilodrostat (LCI699) | -21.20% | 1.12% | | 7.34% | | 12.35% | | |
| Hormone_Safinamide Mesylate | -0.03% | 4.86% | | 1.15% | | 20.08% | | |
| Hormone_Rasagiline Mesylate | -3.37% | 0.32% | | 11.28% | | 2.63% | | |
| Hormone_Paeonol | -1.95% | -0.88% | | 7.26% | | 1.06% | | |
| Hormone_Moclobemide | -0.71% | 8.15% | | 20.00% | | -5.12% | | |
| Hormone_Pargyline hydrochloride | -2.21% | 4.46% | | 18.54% | | -3.13% | | |
| Hormone_Tranylcypromine (2-PCPA) HCl | -10.33% | 2.67% | | 3.96% | | 5.92% | | |
| Hormone_Isatin | -15.33% | -4.08% | | 1.37% | | -6.41% | | |
| Hormone_Iproniazid | -12.67% | -1.27% | | 2.32% | | 2.43% | | |
| Hormone_Safinamide | 7.00% | 0.28% | | 9.83% | | 23.97% | | |
| Hormone_Rasagiline | -5.00% | 5.50% | | 19.08% | | 5.78% | | |
| Hormone_Hypericin | 91.33% | 19.16% | | 93.29% | | 81.30% | | |
| Hormone_Sennoside A | 25.33% | -6.34% | | 26.23% | | 62.97% | | |
| Hormone_Abiraterone Acetate | 11.24% | -21.41% | | -19.94% | | 72.45% | | |
| Hormone_Ketoconazole | 95.07% | 44.17% | | 64.12% | | 78.83% | | |
| Hormone_Tolcapone | 7.41% | 0.33% | | 4.65% | | -40.15% | | |
| Hormone_Trilostane | -17.27% | -3.98% | | -6.98% | | -12.16% | | |
| Lipid_Torcetrapib | 7.00% | -27.49% | | 4.05% | | 19.09% | | |
| Lipid_Isoniazid | 5.33% | 4.01% | | 6.92% | | 10.16% | | |
| Lipid_Lithocholic acid | -6.67% | -7.43% | | -3.73% | | 31.25% | | |
| Lipid_Pitavastatin Calcium | -18.33% | -28.29% | | -8.42% | | -18.16% | | |
| Lipid_Simvastatin | 52.00% | -7.65% | | 19.08% | | 92.06% | | |
| Lipid_Fluvastatin Sodium | -16.33% | -22.74% | | 2.08% | | -19.35% | | |
| Lipid_Lovastatin | 3.36% | -8.36% | | -1.65% | | -8.08% | | |
| Lipid_Atorvastatin Calcium | -2.67% | -21.84% | | 7.20% | | 0.20% | | |
| Lipid_Rosuvastatin Calcium | -24.00% | 5.71% | | -14.22% | | 42.56% | | |
| Lipid_Clinofibrate | -14.67% | 4.37% | | -12.10% | | -0.52% | | |
| Lipid_Pravastatin sodium | -6.00% | 8.15% | | 2.82% | | 8.30% | | |
| Lipid_Mevastatin | -17.00% | 6.85% | | 7.34% | | -13.21% | | |
| Lipid_Pravastatin | 2.33% | 9.93% | | 3.01% | | 16.88% | | |
| Lipid_atorvastatin | -21.33% | -8.31% | | 7.62% | | -3.64% | | |
| Lipid_Ezetimibe | 17.06% | 8.28% | | 7.62% | | 84.63% | | |
| Lipid_Probucol | -11.27% | 8.72% | | 0.11% | | -12.93% | | |
| Lipid_Tanshinone IIA | 18.90% | -9.86% | | 6.89% | | 17.08% | | |
| Lipid_Cetilistat | 21.75% | -3.64% | | 23.31% | | 49.38% | | |
| Lipid_Orlistat | 16.73% | 9.14% | | 10.63% | | 62.43% | | |
| Lipid_Zileuton | 4.88% | 9.07% | | 14.32% | | -9.65% | | |
| Lipid_Nordihydroguaiaretic acid | 2.15% | -9.67% | | 48.62% | | 70.06% | | |
| Lipid_Esculetin | 3.07% | -3.14% | | 10.10% | | 46.80% | | |
| Lipid_Tanshinone I | 39.31% | 17.25% | | -9.17% | | 2.08% | | |
| Lipid_Polydatin | 21.22% | 31.88% | | 1.61% | | 11.00% | | |
| Lipid_Halobetasol Propionate | -3.28% | 2.34% | | 46.43% | | 71.41% | | |
| Lipid_Clofazimine | 86.01% | 54.99% | | 82.97% | | 86.08% | | |
| Lipid_Quinacrine | 96.63% | 95.35% | | 93.30% | | 86.94% | | |
| Lipid_Trigonelline | -0.61% | 24.48% | | 6.05% | | -3.51% | | |
| Lipid_Gemfibrozil | -4.48% | 14.97% | | -4.29% | | -4.88% | | |
| Lipid_Fenofibrate | 0.02% | 59.82% | | 1.41% | | 32.86% | | |
| Lipid_Rosiglitazone HCl | -5.50% | 43.22% | | -15.22% | | -4.05% | | |
| Lipid_Rosiglitazone maleate | -2.74% | 39.84% | | -11.29% | | -4.67% | | |
| Lipid_Rosiglitazone | -6.97% | 8.06% | | -14.86% | | 5.66% | | |
| Lipid_Pioglitazone | 0.43% | 12.40% | | -6.44% | | 3.07% | | |
| Lipid_Bezafibrate | -14.47% | -6.87% | | -9.99% | | 28.84% | | |
| Lipid_Clofibric Acid | 4.62% | -4.61% | | -8.78% | | 17.48% | | |
| Lipid_Fenofibric acid | -9.51% | -8.92% | | -6.46% | | -61.50% | | |
| Lipid_Choline Fenofibrate | 11.11% | -8.71% | | 0.46% | | 0.28% | | |
| Lipid_L-Cycloserine | 17.84% | -10.00% | | 7.92% | | -55.21% | | |
| Lipid_Nicotinic Acid | 1.89% | 1.00% | | 9.66% | | -20.31% | | |
| Metabolic transport_Methyldopa | 50.61% | 5.00% | | 40.86% | | 68.26% | | |
| Metabolic transport_Topiramate | 9.15% | -1.19% | | -0.26% | | -2.05% | | |
| Metabolic transport_Cyclofenil | 20.58% | 5.63% | | 10.64% | | 43.46% | | |
| Metabolic transport_Triamcinolone Acetonide | -15.67% | -5.82% | | 13.07% | | 28.83% | | |
| Metabolic transport_Ranitidine | -25.33% | 5.93% | | -3.36% | | 8.40% | | |
| Metabolic transport_Benzbromarone | -4.27% | -1.67% | | 3.24% | | 70.46% | | |
| Metabolic transport_Cilostazol | 34.25% | 44.95% | | 2.45% | | 20.04% | | |
| Metabolic transport_Nonivamide | 1.26% | -2.67% | | 5.96% | | -28.49% | | |
| Metabolic transport_Amprolium HCl | -13.67% | 8.67% | | 6.50% | | 39.81% | | |
| Nucleotide_Leflunomide | 24.17% | -18.26% | | 4.78% | | 12.46% | | |
| Nucleotide_Metabolic transport_Cyclofenil | 41.39% | -6.25% | | 18.02% | | 21.30% | | |
| Nucleotide_Gimeracil | 12.38% | 7.48% | | 5.97% | | -7.77% | | |
| Nucleotide_Mycophenolic acid | 14.00% | -5.51% | | 15.98% | | 2.26% | | |
| Nucleotide_Brequinar | -11.05% | -22.64% | | -3.66% | | -19.41% | | |
| Nucleotide_Teriflunomide | 1.55% | -18.91% | | -7.91% | | 7.97% | | |
| Redox_Methocarbamol | -2.42% | -5.03% | | 0.95% | | -7.77% | | |
| Redox_Tioxolone | -14.20% | 2.39% | | -4.96% | | -12.23% | | |
| Redox_Brinzolamide | -5.54% | -4.40% | | -1.53% | | -11.42% | | |
| Redox_Methazolamide | -3.14% | 1.65% | | -2.31% | | -66.12% | | |
| Redox_Acetazolamide | 18.84% | 1.18% | | 1.73% | | -77.25% | | |
| Redox_Benzenesulfonamide | -10.55% | 4.03% | | 1.74% | | -69.58% | | |
| Redox_Dorzolamide HCl | -7.67% | 0.52% | | -2.90% | | -84.50% | | |
| Redox_Metyrapone | 20.25% | 8.31% | | 6.68% | | -21.08% | | |
| Redox_Disulfiram | 24.82% | 44.96% | | 70.98% | | -10.68% | | |
| Redox_Fomepizole | 3.78% | -1.66% | | 4.40% | | -5.03% | | |
| Redox_Daidzin | 23.45% | 4.94% | | 9.61% | | -4.44% | | |
| Redox_Emodin | 14.33% | 11.83% | | 8.96% | | -6.19% | | |
| Redox_Naringin | -5.89% | 1.33% | | -3.43% | | 6.64% | | |
| Redox_Naringin Dihydrochalcone | -13.01% | 7.36% | | 0.51% | | 9.27% | | |
| Redox_Sodium Danshensu | -18.29% | -4.07% | | 17.83% | | 82.71% | | |
| Redox_Danshensu | 10.87% | -14.89% | | -5.69% | | 75.63% | | |
| Redox_Vitamin C | 1.70% | -1.16% | | 5.85% | | -5.52% | | |
| Redox_Vitamin E Acetate | 1.50% | 20.72% | | 6.11% | | -10.23% | | |
| Redox_(±)-α-Tocopherol | -11.33% | 9.91% | | 0.68% | | -51.85% | | |
| Redox_Sodium ascorbate | 0.33% | 3.22% | | 3.50% | | 55.50% | | |
| Vitamin_Isotretinoin | -1.36% | 2.79% | | -14.22% | | 57.67% | | |
| Vitamin_Tocofersolan | 12.67% | 17.00% | | 9.05% | | 9.10% | | |
| Vitamin_Adapalene | 10.00% | -33.79% | | 21.47% | | 74.64% | | |
| Vitamin_Acitretin | 83.96% | 3.00% | | -2.73% | | 50.28% | | |
| Vitamin_Tazarotene | 49.33% | -19.10% | | -5.53% | | 26.56% | | |
| Vitamin_Tretinoin | -1.89% | -9.76% | | 9.25% | | 31.18% | | |
| Vitamin_Bexarotene | 26.67% | 3.67% | | 3.01% | | 58.11% | | |
| Vitamin_Tamibarotene | -4.22% | 7.30% | | 8.43% | | 42.14% | | |
| Vitamin_Etretinate | 15.32% | 17.34% | | 5.14% | | 54.50% | | |
| Vitamin_Calcitriol | -0.31% | 7.00% | | 7.09% | | 52.86% | | |
| Vitamin_Doxercalciferol | -7.52% | -6.00% | | 14.31% | | 85.01% | | |
| Vitamin_Alfacalcidol | 0.91% | 5.00% | | 9.02% | | 86.21% | | |
| Vitamin_Phenindione | 1.79% | -9.00% | | 4.31% | | -20.49% | | |
| Vitamin_Biotin (Vitamin B7) | -1.03% | 5.87% | | 8.54% | | -15.71% | | |
| Vitamin_cholecalciferol (Vitamin D3) | 33.17% | -6.88% | | 29.37% | | 82.37% | | |
| Vitamin_Vitamin A Acetate | 13.67% | 3.26% | | 13.12% | | -14.98% | | |
| Vitamin_Vitamin K2 | 6.24% | -3.53% | | 12.91% | | -38.80% | | |
| Vitamin_Beta Carotene | 46.67% | 6.63% | | 16.65% | | 60.79% | | |
| Vitamin_Retinyl (Vitamin A) Palmitate | 46.67% | 0.99% | | 26.11% | | 69.35% | | |
| Others_Apixaban | 21.34% | 12.56% | | -7.45% | | 30.63% | | |
| Others_Rivaroxaban | -3.67% | 10.61% | | 21.27% | | 49.81% | | |
| Others_Edoxaban | 0.67% | 11.14% | | 2.64% | | 39.32% | | |
| Others_Methoxsalen | 1.59% | 5.00% | | 3.54% | | 24.64% | | |
| Others_Ozagrel HCl | -10.26% | -2.00% | | 0.24% | | 9.19% | | |
| Others_Piperine | -9.43% | -4.00% | | -1.22% | | 20.44% | | |
| Others_Diosmetin | -4.82% | 17.00% | | -18.09% | | 34.86% | | |
| Others_Ozagrel | -6.18% | 3.98% | | -2.31% | | 11.63% | | |
| Others_Cobicistat (GS-9350) | -5.85% | 32.04% | | 0.23% | | 55.06% | | |
| Others_Chlorzoxazone | -15.75% | 3.68% | | 9.49% | | -0.26% | | |
| Others_Rolipram | 19.95% | 38.27% | | -3.73% | | 7.34% | | |
| Others_Sildenafil Citrate | 3.26% | 9.92% | | -4.36% | | 19.72% | | |
| Others_Dyphylline | 10.53% | -10.33% | | -8.13% | | 6.43% | | |
| Others_Tadalafil | 12.41% | 8.04% | | -14.13% | | -3.58% | | |
| Others_Pimobendan | 24.68% | 31.30% | | -4.42% | | 21.72% | | |
| Others_Aminophylline | 24.26% | 27.68% | | -9.22% | | 7.77% | | |
| Others_Dipyridamole | 26.11% | 55.56% | | 7.45% | | 62.75% | | |
| Others_S- (+)-Rolipram | 9.74% | 7.67% | | -11.41% | | 0.22% | | |
| Others_Roflumilast | 8.32% | -8.21% | | -14.00% | | -5.32% | | |
| Others_Luteolin | 34.95% | 28.39% | | -13.20% | | 59.97% | | |
| Others_Milrinone | 26.90% | 35.93% | | -6.39% | | 6.31% | | |
| Others_Vardenafil HCl Trihydrate | 25.73% | 37.49% | | -4.60% | | -3.24% | | |
| Others_Anagrelide HCl | 8.23% | 27.34% | | -6.22% | | 6.25% | | |
| Others_Avanafil | 22.34% | 37.84% | | -2.68% | | 58.17% | | |
| Others_Fenspiride HCl | 5.94% | 35.07% | | -8.26% | | -2.61% | | |
| Others_Doxofylline | -3.71% | 22.55% | | -8.60% | | -4.08% | | |
| Others_Sildenafil Mesylate | -0.64% | 23.45% | | -3.95% | | -13.32% | | |
| Others_Ibudilast | 5.10% | 25.44% | | 3.02% | | 0.52% | | |
| Others_Crisaborole (AN2728) | 21.71% | 35.62% | | 8.34% | | 23.60% | | |
| Others_Udenafil | 15.62% | 28.12% | | 12.49% | | 84.95% | | |
| Others_Sildenafil | 13.37% | 26.36% | | -9.70% | | -20.73% | | |
| Others_Sodium stibogluconate | -0.98% | 29.48% | | -18.39% | | -16.59% | | |
| Others_Phytol | 14.13% | 0.18% | | 1.40% | | -2.99% | | |
| Others_Apremilast (CC-10004) | 16.35% | 8.00% | | 7.18% | | -29.36% | | |
| Others_Tipifarnib | 2.36% | 60.00% | | 70.52% | | 81.11% | | |

**Table S2. Baseline characteristics of samples for single-cell RNA sequencing Group2.**

| **Sample** | **Sex** | **Age(y)** | **Diagnosis** | **State** | **Site of sample** | **Remarks** |
| --- | --- | --- | --- | --- | --- | --- |
| P1 | Female | 51 | AITL | ND | Peripheral blood | Newly diagnosed AITL, untreated |
| P2 | Male | 31 | AITL | ND | Lymph node | Newly diagnosed AITL, untreated |
| P3 | Male | 55 | AITL | ND | Lymph node | Newly diagnosed AITL, untreated |
| P4 | Male | 56 | AITL | RR | Peripheral blood | Recurrence, Day146 post CHOPE*2、Chidamide+GOD*2, C4D146 |
| P5 | Male | 56 | AITL | RR | Peripheral blood | Recurrence, Day42 post CHOPE*4, C4D42 |
| P6 | Male | 67 | AITL | RR | Peripheral blood | Recurrence, Day30 post Azacitidine +CHOP*2, C2D30 |
| P7 | Male | 55 | AITL | RR | Lymph node | Recurrence, Day41 post CHOPE*2, C2D41 |
| N1 | Female | 50 | NC | / | Peripheral blood | / |
| N2 | Male | 30 | NC | / | Peripheral blood | / |
| N3 | Male | 55 | NC | / | Peripheral blood | / |
| N4 | Male | 56 | NC | / | Peripheral blood | / |
| N5 | Male | 62 | NC | / | Peripheral blood | / |

NC, normal control; AITL, angioimmunoblastic T cell lymphoma; ND, newly diagnosed; RR, relapsed/refractory; CHOP/CHOPE, cyclophosphamide, doxorubicin, vincristine, and prednisolone/plus etoposide; GOD, gemcitabine, oxaliplatin, dexamethasone; CxDy, x represents the number of therapeutic cycles; y represents the date of lymph node biopsy calculated from the start of therapy.

**Table S3. Clinical and pathological characteristics of samples in the tissue microarray.**

| Code number | Gender | Age | Primary site | Diagnosis |
| --- | --- | --- | --- | --- |
| D07A0359 | Female | 58 | tonsil | RLH |
| D07A0360 | Male | 29 | tonsil | RLH |
| D07A0361 | Female | 64 | tonsil | RLH |
| D07A0362 | Male | 28 | tonsil | RLH |
| D07A0363 | Male | 46 | tonsil | RLH |
| D07A0364 | Male | 34 | tonsil | RLH |
| D07A0365 | Female | 20 | tonsil | RLH |
| D07A0366 | Female | 7 | tonsil | RLH |
| H02A0047 | Female | 73 | inguinal lymph nodes | AITL |
| H02A0166 | Female | 87 | cervical lymph nodes | AITL |
| H02A0169 | Male | 63 | cervical lymph nodes | AITL |
| H99A0049 | Female | 68 | mesenteric lymph nodes | ALCL |
| H02A0051 | Male | 41 | Inguinal lymph nodes | ALCL |
| H99A0010 | Male | 51 | occipital lymph nodes | ALCL |
| H99A0601 | Female | 52 | retroauricular lymph nodes | CD8+ PCAL |
| D09A4462 | Male | 61 | stomach | ENKTL |
| H99A0052 | Male | 55 | stomach | ENKTL |
| E01A0136 | Male | 27 | nasal cavity | ENKTL |
| E01A0142 | Female | 52 | nasal cavity | ENKTL |
| H02A0042 | Female | 73 | Inguinal lymph nodes | ENKTL |
| H02A0048 | Male | 53 | Inguinal lymph nodes | ENKTL |
| H99A0013 | Female | 23 | skin | ENKTL |
| H99A0376 | Female | 72 | small intestine | MEITL |
| H99A0240 | Male | 55 | small intestine | MEITL |
| H99A0001 | Male | 75 | lower limb lymph node | MF |
| D09A0943 | Female | 74 | stomach | PTCL-NOS |
| H99A0166 | Male | 57 | stomach | PTCL-NOS |
| H99A0068 | Male | 49 | ileocecal lymph nodes | PTCL-NOS |
| H99A0375 | Male | 36 | ileocecal lymph nodes | PTCL-NOS |
| D99A0122 | Male | 62 | small intestine | PTCL-NOS |
| H99A0048 | Male | 34 | small intestine | PTCL-NOS |
| H99A0134 | Male | 49 | small intestine | PTCL-NOS |
| H02A0043 | Male | 41 | cervical lymph node | PTCL-NOS |
| H02A0044 | Male | 75 | axillary lymph node | PTCL-NOS |
| H02A0045 | Male | 38 | axillary lymph node | PTCL-NOS |
| H02A0046 | Male | 20 | cervical lymph node | PTCL-NOS |
| H02A0164 | Male | 90 | cervical lymph node | PTCL-NOS |
| H02A0167 | Female | 81 | axillary lymph node | PTCL-NOS |
| H02A0168 | Female | 81 | axillary lymph node | PTCL-NOS |
| H02A0504 | Female | 68 | cervical lymph node | PTCL-NOS |
| H99A0056 | Male | 72 | lower limb lymph node | PTCL-NOS |
| H99A0011 | Female | 87 | axillary lymph node | SPTCL |
| H99A0053 | Female | 38 | epitrochlear nodes | SPTCL |

RLH, reactive lymphoid hyperplasia; AITL, angioimmunoblastic T cell lymphoma; ALCL, anaplastic lymphoma kinase positive anaplastic large cell lymphoma; PCAL, primary cutaneous acral lymphoma; ENKTL, extranodal NK/T-cell lymphoma; MEITL, monomorphic epitheliotropic intestinal T-cell lymphoma; MF, mycosis fungoides; PTCL-NOS, PTCL-not otherwise specified; SPTCL, subcutaneous panniculitic T-cell lymphoma.

**Table S4. Sequences of primers and sgRNAs used in this study.**

|  | Name | Sequence | Source |
| --- | --- | --- | --- |
| sgRNAs | sgControl | ATTTCGCAGATCATCGACAT | Brunello Library |
|  | sgSLC3A2#1 | CTGGAAAGATATACCAGTAT | Brunello Library |
|  | sgSLC3A2#2 | CTTGTTGTTAGAGGAGCAAG | Brunello Library |
|  | sgJUNB#1 | AAAACAGCGTCCGCGCACTG | Brunello Library |
|  | sgJUNB#2 | ATCGGTGATTCTGCAAGTAA | Brunello Library |
|  | sgSSRP1#1 | GGATGCTGTGATCATCCTGG | Brunello Library |
|  | sgSSRP1#2 | ATAGAAACGCTCATCTCCCG | Brunello Library |
| siRNAs | ATF3-F | GAGAAACCUCUUUAUCCAATT | In this study |
|  | ATF3-R | UUGGAUAAAGAGGUUUCUCTT | In this study |
|  | ATF4-F | UUGGCUCGCUGUGGACAGUUUA | In this study |
|  | ATF4-R | UAAACUGUCCACAGCGAGCCAA | In this study |
|  | ATF5-F | CACCUGACCUGGAAGCUAUTT | In this study |
|  | ATF5-R | AUAGCUUCCAGGUCAGGUGTT | In this study |
|  | CEBPB-F | CCUCGCAGGUCAAGAGCAATT | In this study |
|  | CEBPB-R | UUGCUCUUGACCUGCGAGGTT | In this study |
|  | CEBPG-F | GGAAUUAGUGUUAUCCAUATT | In this study |
|  | CEBPG-R | UAUGGAUAACACUAAUUCCTT | In this study |
|  | ELF1-F | GCUGAGGCACUCCUCAAUATT | In this study |
|  | ELF1-R | UAUUGAGGAGUGCCUCAGCTT | In this study |
|  | KLF2-F | GCACCGACGACGACCUCAATT | In this study |
|  | KLF2-R | UUGAGGUCGUCGUCGGUGCTT | In this study |
|  | KLF10-F | GAACAAAACUGCAGAGAAATT | In this study |
|  | KLF10-R | UUUCUCUGCAGUUUUGUUCTT | In this study |
|  | JUNB-F | CGACUACAAACUCCUGAAATT | In this study |
|  | JUNB-R | UUUCAGGAGUUUGUAGUCGTT | In this study |
| qRT-PCR | SLC3A2-F | CGTGTCATTCTGGACCTTACTC | In this study |
|  | SLC3A2-R | AAACTCCAGAGCATCCTTCAC | In this study |
|  | ATF3-F | CTGGAAAGTGTGAATGCTGAAC | In this study |
|  | ATF3-R | ATTCTGAGCCCGGACAATAC | In this study |
|  | ATF4-F | GGAGATAGGAAGCCAGACTACA | In this study |
|  | ATF4-R | GGCTCATACAGATGCCACTATC | In this study |
|  | ATF5-F | GTCTATGCCCGTCACATAACA | In this study |
|  | ATF5-R | CCAGACAACCACCTGTAAGAA | In this study |
|  | CEBPB-F | TCCAAACCAACCGCACAT | In this study |
|  | CEBPB-R | AGAGGGAGAAGCAGAGAGTTTA | In this study |
|  | CEBPG-F | TAAGAGTCGTGAGGTCCTTCT | In this study |
|  | CEBPG-R | CTATCCTGTCTCCTGGCTCTAT | In this study |
|  | ELF1-F | ACACAGCAGGTGCAAGAA | In this study |
|  | ELF1-R | CTGGTCGTGGTGGTTTAGTT | In this study |
|  | JUNB-F | TCTACCACGACGACTCATACA | In this study |
|  | JUNB-R | GGCTCGGTTTCAGGAGTTT | In this study |
|  | KLF10-F | CCTTCTGACTTTGAACCCTCTC | In this study |
|  | KLF10-R | AATGTGAGGTTTGGCAGTATCT | In this study |
|  | KLF2-F | AGCTGAGAGGTGTTTGGTAATC | In this study |
|  | KLF2-R | GAAGTGAGGCCAGGCTATTT | In this study |
| CHIP-qPCR | SLC3A2-F | GCATTGCGGCTTGGTTTTCTC | In this study |
|  | SLC3A2-R | CAGCTCAGAACAGGGTTAGAG | In this study |

**Data S1. Key resources table**

| **Reagent or resource** | **Source** | **Identifier** |
| --- | --- | --- |
| **Antibodies** |  |  |
| Rabbit-anti-SLC3A2 | Proteintech | Cat# 15193-1-AP; RRID: AB_2254909 |
| Rabbit-anti-SLC7A7 | Proteintech | Cat# 26403-1-AP |
| Rabbit-anti-SLC7A1 | Invitrogen | Cat# PA5-42426; RRID:AB_2608995 |
| Mouse anti-ASS1 | Santa Cruz Biotechmology | Cat# sc-514726 |
| Mouse anti-ASL | Santa Cruz Biotechmology | Cat# sc-374353; RRID:AB_10990314 |
| Mouse anti-ARG2 | Santa Cruz Biotechmology | Cat# sc-393496; RRID: AB_2890065 |
| Mouse anti-OTC | Santa Cruz Biotechmology | Cat# sc-515791 |
| Rabbit anti-GAMT | Proteintech | Cat# 10880-1-AP; RRID: AB_2109304 |
| Rabbit anti-AGMAT | Proteintech | Cat# 21899-1-AP; RRID: AB_3085677 |
| Rabbit anti-SRM | Proteintech | Cat# 19858-1-AP; RRID: AB_10665555 |
| Rabbit anti-FLAG | ABclonal | Cat# AE092; RRID: AB_2940847 |
| Rabbit anti-PARP | Cell Signaling Technology | Cat# 9542; RRID: AB_2160739 |
| Mouse anti-JUNB | Santa Cruz Biotechmology | Cat# sc-398061 |
| Rabbit anti total OXPHS | Abcam | Cat# ab110411; RRID: AB_2756818 |
| Rabbit anti-SSRP1 | Proteintech | Cat# 15696-1-AP; RRID: AB_2195756 |
| Rabbit anti-SSRP1 | Cell Signaling Technology | Cat# 13421; RRID: AB_2714160 |
| Rabbit anti-Ki-67 | Proteintech | Cat# 27309-1-AP; RRID: AB_2756525 |
| Rabbit anti-Cleaved-caspase3 | Proteintech | Cat# 25128-1-AP; RRID: AB_3073913 |
| Mouse anti-β-Actin | Proteintech | Cat# 60008-1-lg |
| Rabbit anti-β-Tublin | Cell Signaling Technology | Cat# 2128; RRID: AB_823664 |
| Mouse anti-GAPDH | Cell Signaling Technology | Cat# 97166; RRID: AB_2756824 |
| PE anti-human CD366 (Tim-3) | Biolegend | Cat# 345005; RRID: AB_1877236 |
| 7-AAD | BD Pharmingen | Cat# 559925; RRID: AB_2869266 |
| APC anti-human CD279 (PD-1) | Biolegend | Cat# 379207; RRID: AB_2922606 |
| APC-Cy™7 anti-human CD8 | BD Pharmingen | Cat# 557760 |
| PC7 anti-human CD3 | Beckman Coulter | Cat# 737657; RRID: AB_2636813 |
| Rabbit anti-CD4 | Cell Signaling Technology | Cat# 93518 |
| Rabbit anti-CD8 alpha | Abcam | Cat# 237709; RRID: AB_2892677 |
| Rabbit anti-Granzyme B | Abcam | Cat# 255598; RRID: AB_2860567 |
| Anti-rabbit IgG, HRP-linked | Cell Signaling Technology | Cat# 7074; RRID: AB_2099233 |
| Anti-mouse IgG, HRP-linked | Cell Signaling Technology | Cat# 7076; RRID: AB_330924 |
| Goat Anti-Mouse lgG-Alexa Fluor 488 | Abmart | Cat# M21011 |
| Goat Anti-Rabbit lgG-Alexa Fluor 594 | Abmart | Cat# M21014 |

| **Biological samples** |  |  |
| --- | --- | --- |
| Paraffin AITL sections | Jiangsu Province Hospital | N/A |
| Paraffin PTCL sections | Shanghai Outdo Biotech | HLymT060PT01 |
| Fresh PTCL samples | Jiangsu Province Hospital | Table S2 |
| Fresh peripheral blood samples | Jiangsu Province Hospital | N/A |

| **Chemicals** |  |  |
| --- | --- | --- |
| Metabolic inhibitor library | Selleck | Table S1 |
| Quinacrine | Sigma-Aldrich | Cat# Q3251 |
| CBL0137 | Selleck | Cat# S8483 |
| Chidamide | Selleck | Cat# S8567 |
| Ketoconozle | Selleck | Cat# S1353 |
| Clofazimine | Selleck | Cat# S4107 |
| Puromycin | Invivogen | Cat# ant-pr-1 |
| Hygromycin | Invivogen | Cat# ant-hg-1 |
| Blasticidin | Invivogen | Cat# ant-bl-05 |
| Lipo3000 | Thermo Fisher Scientific | Cat# L3000015 |
| Recombinant Human IL-2 | BD Pharmingen | Cat# 554603 |
| Human CD3/CD28 T Cell Activator | STEMCELL Technologies | Cat# 10971 |
| CFSE | Absin | Cat# abs9106 |
| MitoSOX red | MedChemExpress | Cat# HY-D055 |
| DAPI | Beyotime | Cat# C1002 |
| Arachidonic acid | MedChemExpress | Cat# HY-109590 |
| L-arginine | Sigma-Aldrich | Cat# 11009 |
| Creatine | MedChemExpress | Cat# HY-W010388 |
| Ornithine | MedChemExpress | Cat# HY-W017018 |
| Putrescine | Sigma-Aldrich | Cat# 51799 |
| Spermidine | MedChemExpress | Cat# HY-B1776 |
| Spermine | MedChemExpress | Cat# HY-B1777 |
| Arg-free diet | Jiangsu XieTong Pharmaceutical Bio-Engineering | XTAA |

| **Critical commercial assays** |  |  |
| --- | --- | --- |
| Annexin V-APC/PI Apoptosis Detection Kit | Vazyme | Cat# A214-01 |
| Annexin V-FITC/PI Apoptosis Detection Kit | Vazyme | Cat# A211-01 |
| Cell Counting Kit-8 solution | Dojindo | Cat# CK04 |
| Arginine Assay Kit | Abcam | Cat# ab252892 |
| Cell Cycle Staining Kit | Liankebio | Cat# CCS012 |
| BCA Protein Assay Kit | Thermo Fisher Scientific | Cat# A55864 |
| Sonication ChIP Kit | ABclonal | Cat# RK20258 |
| Immunoprecipitation Kit with Protein A+G Agarose Gel | Beyotime | Cat# P2197M |
| Seahorse XF Cell Mito Stress Test Kit | Agilent Technologies | Cat# 10315-100 |
| ATP Assay Kit | Beyotime | Cat# S0026 |
| CD3 microbeads, human | Miltenyi | Cat# 130-050-101 |
| Hiscript III RT SuperMix for qPCR (+gDNA wiper) | Vazyme | Cat# R323-01 |
| Taq Pro Universal SYBR qPCR Master Mix | Vazyme | Cat# Q712-02 |
| QIAprep Spin Miniprep Kit | Qiagen | Cat# 27106 |

| **Deposited data** |  |  |
| --- | --- | --- |
| Data files for bulk RNA-seq | This study | GEO: GSE160119 and GSE58445 |
| Data files for scRNA-seq | This study | GSA for human: HRA004525 |

| **Experimental models: Cell lines** |  |  |
| --- | --- | --- |
| HEK293T | ATCC | CRL-3216 |
| Human: Karpas 299 | Cobioer | CBP60271; RRID:CVCL_1324 |
| Human: SU-DHL-1 | BNCC | BNCC342094; RRID:CVCL_0063 |
| Human: H9 | BNCC | BNCC339983; RRID:CVCL_1240 |
| Human: Hut78 | BNCC | BNCC359882; RRID:CVCL_0337 |
| Human: MT-4 | BNCC | BNCC338052; RRID:CVCL_2632 |

| **Experimental models: Organisms/strains** |  |  |
| --- | --- | --- |
| NCG mice | Gempharmatech Company | T001475; RRID:IMSR_CRL:572 |

| **Oligonucleotides** |  |  |
| --- | --- | --- |
| List of oligonucleotides | This study | Table S4 |

| **Recombinant DNA** |  |  |
| --- | --- | --- |
| pLentiGuide-Puro | Addgene | 52963; RRID:Addgene_174326 |
| psPAX2 | Addgene | 12260; RRID:Addgene_12260 |
| pMD2.G | Addgene | 12259; RRID:Addgene_12259 |
| pCDH-SLC3A2 | This study | N/A |

| **Software and algorithms** |  |  |
| --- | --- | --- |
| GraphPad Prism v9.0 | GraphPad Software | [www.graphpad.com;](http://www.graphpad.com;) RRID:SCR_002798 |
| SPSS Statistics v26.0 | IBM | <https://www.spss.cm.cn;> RRID:SCR_002865 |
| R v4.0.0 | R Project | <https://www.r-project.org;> RRID:SCR_001905 |
| FlowJo v10 | FlowJo Software | [www.flowjo.com;](http://www.flowjo.com;) RRID:SCR_008520 |
| Biorender | Biorender | <https://biorender.com/;> RRID:SCR_018361 |
| MetaboAnalyst 5.0 | MetaboAnalyst | <https://www.metaboanalyst.ca/;> RRID:SCR_015539 |
| CeleScope v1.9.0 | Singleron | <https://github.com/singleron-RD/CeleScope;> RRID:SCR_023553 |
